# Stable isotopes reveal opportunistic foraging in a spatiotemporally heterogeneous environment: Bird assemblages in mangrove forests

**DOI:** 10.1371/journal.pone.0206145

**Published:** 2018-11-15

**Authors:** Christina A. Buelow, April E. Reside, Ronald Baker, Marcus Sheaves

**Affiliations:** 1 College of Science and Engineering, James Cook University, Townsville, Queensland, Australia; 2 Centre for Tropical Water & Aquatic Ecosystem Research (TropWATER), James Cook University, Townsville, Queensland, Australia; 3 Centre for Biodiversity and Conservation Science, The University of Queensland, Brisbane, Queensland, Australia; 4 Dauphin Island Sea Lab, The University of South Alabama, Dauphin Island, Alabama, United States; Phillip Island Nature Parks, AUSTRALIA

## Abstract

Environmental heterogeneity can foster opportunistic foraging by mobile species, resulting in generalized resource and habitat use. Determining species’ food web roles is important to fully understand how ecosystems function, and stable isotopes can provide insight into the foraging ecology of bird assemblages. We investigated flexibility of food choice in mangrove bird assemblages of northeast Australia by determining whether species’ carbon and nitrogen isotopic values corresponded to foraging group classification described in the literature, such as groups of species that are omnivorous or insectivorous. Subsequently, we evaluated foraging group isotopic niche size, overlap, degree of individual specialisation, and the probable proportions of coastal resources that contribute to their collective diets. We found that mangrove birds are more opportunistic when foraging than expected from previous diet studies. Importantly, relationships between the dietary diversity of species within a foraging group and isotopic niche size are spatially inconsistent, making inferences regarding foraging strategies difficult. However, quantifying individual specialisation and determining the probable relative contributions of coastal resources to the collective diet of isotope-based foraging groups can help to differentiate between specialised and generalised foraging strategies. We suggest that flexibility in mangrove bird foraging strategy occurs in response to environmental heterogeneity. A complementary approach that combines isotopic analysis with other dietary information (collated from previous diet studies using visual observation or gut content analyses) has provided useful insight to how bird assemblages partition resources in spatiotemporally heterogeneous environments.

## Introduction

Opportunistic foraging has been observed in many avian taxa, including insectivorous and nectivourous passerines [1, 2, 3], waterbirds [4], raptors [5], and seabirds [6]. This often occurs when resource availability is patchy and unpredictable across space and time, making flexibility in food choice a means for survival. For example, shorebirds are known to forage opportunistically when fluctuating water levels at wetland stopover sites cause high variability in prey abundance [7, 8], and when tides or floodwaters inundate their preferred foraging areas [9, 10]. Therefore, in environments characterized by heterogeneous resource availability, opportunism can occur across multiple features of a species’ ecological niche, resulting in generalized resource and habitat choice. Given the importance of species’ food web roles for ecosystem functioning, there is a need to understand relationships between environmental heterogeneity and the niches that species are able to occupy.

Heterogeneity can manifest from the abiotic or biotic characteristics of an environment. For example, coastal mangrove forests are located at the land-sea interface, and their functioning is influenced by abiotic factors, such as tides, and biotic factors, such as the extent and configuration of adjacent vegetation. For forest birds, tidal inundation means that the availability of many mangrove resources fluctuates daily, suggesting foraging flexibility is likely to be important. Mangroves also offer estuarine prey items (e.g. mudskippers and crabs) that are not found in terrestrial forest types. Furthermore, mangroves are often situated in a complex mosaic of adjacent vegetation types such as grasslands, saltmarshes, and woodlands, and this could mean that flexibility in foraging strategy and choice of foraging habitat may be advantageous for highly mobile forest avifauna.

Relative to other forest types, mangroves support few bird species that are obligate habitat (mangrove) specialists and instead host many species with generalized foraging niches [11, 12, 13]. However, foraging niches have traditionally been examined through visual observation or gut content analyses and, unless individuals can be tracked or recaptured, these methods are inappropriate for determining the consistency of an individual’s foraging strategy. For example, visual observation can indicate what resources are consumed by a species in a specific area, but will not provide information on whether individuals move to forage in different habitats, or if individuals consume different resources over time.

Stable isotope analysis provides complementary information to traditional measurements of foraging ecology. The carbon (^13^C/^12^C) and nitrogen (^15^N/^14^N) stable isotope ratios of resources vary depending on the photosynthetic pathway of primary producers and with trophic level, respectively, and are integrated into the tissues of consumers [14]. Therefore, isotopic values can be used as ‘tracers’ of resources that are consumed, allowing consumer food webs and ‘isotopic niches’ to be constructed if there is sufficient variability in the isotopic values of basal resources [14, 15, 16, 17, 18].

An isotopic niche represents only a subset of a species’ entire ecological niche [15, 18, 19]. Bird carbon isotopic values represent foraging habitat choice where habitats are isotopically distinct (e.g. saltmarsh vs. forest), and nitrogen isotopic values represent resource choice by proxy of trophic level due to enrichment of ^15^N in tissues of individuals that occupy high trophic positions [20]. Also, bird blood and claw tissues differ in how quickly their isotopic values are integrated (i.e. isotopic turnover rate), providing a temporal comparison of individual foraging choice (weeks vs. months) [21, 22]. Therefore, stable isotopes offer a unique way to quantify species’ niches and food-web positions in spatiotemporally heterogeneous environments.

We investigated spatiotemporal opportunism in mangrove birds using carbon and nitrogen stable isotopes to provide insight into their resource use and flexibility. Categorizing species into foraging groups (e.g. carnivores, omnivores, insectivores, or nectarivores) is a useful way to understand how bird assemblages partition resources [23]. However, species employing opportunistic foraging strategies may not necessarily fit within literature-prescribed foraging groups. Therefore, we investigated whether the isotopic values of bird species occupying mangrove forests corresponded to their foraging groups described in the literature, anticipating discordance between literature-based and isotopic bird foraging groups.

Following identification of isotopic foraging groups, we measured their isotopic niche size and overlap [16, 18] expecting patterns indicative of generalized foraging strategies (although see Fig A in the S1 Appendix for an illustration of some challenges in interpreting isotopic niche size). We also used tissues with different isotopic turnover rates to examine seasonal variability in resource selection and individual specialisation, expecting that temporally opportunistic birds will have dissimilar isotopic values between blood and claws [21, 22]. Finally, we constructed the isotopic food web of mangrove bird foraging groups to determine the probable proportions of resources that contribute to their diet. Together, these findings provide insight to the complex resource use of these species and thus the functioning of this system.

## Methods

### Study area

**Fig 1:**
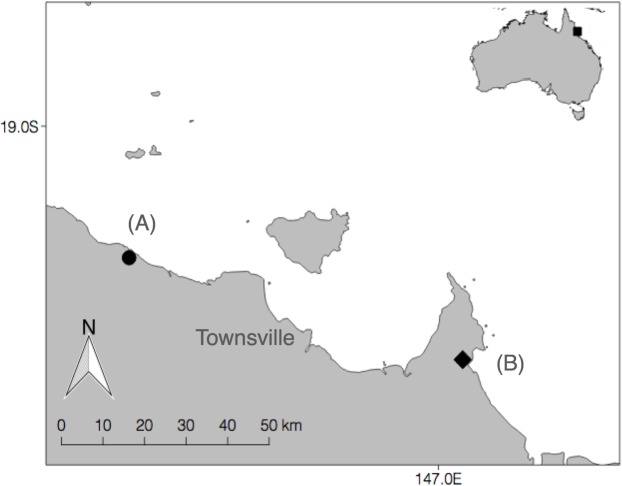
Map displaying two mangrove forest sampling sites in northeast Queensland, Australia. (A) Healy Creek (circle) and (B) Cocoa Creek (diamond).

This study was conducted at two mangrove forest sites in the Brigalow Belt North bioregion of northeast Australia [24] (Fig 1). This region has a dry tropical climate, with rainfall occurring primarily in the wet season (November to April, ~590mm/year). In addition to mangrove forest, coastal vegetation of this region includes: saltmarsh, rainforest, grassland, agricultural land, and Eucalypt, Acacia, and Melaleuca woodlands and forests.

### Bird capture and foraging group classification

This study was carried out under approval from the Animal Welfare and Ethics Committee at James Cook University (Protocol Number: A2147). All animals were handled with extreme care and as efficiently as possible to reduce stress and minimise suffering.

Bird capture and sampling occurred during the months of March-April, 2015 (wet season) and July-August, 2015 (dry season) at Cocoa Creek (Fig 1A), and only during the wet season months at Healy Creek (Fig 1B). These locations were chosen for their accessibility and for their habitat composition (i.e. a mosaic of mangrove, saltmarsh, and terrestrial forest habitat types). Eight to ten nylon mist-nets (dimensions = 12 × 2.5 m, mesh-size = 16 mm) were used to catch birds on each day of sampling, targeting primarily passerine bird species (see Table A in the S2 Appendix for a full species list). Locations for mist-nets were chosen haphazardly at each site, and shifted approximately every two days. Nets were opened before dawn and closed by 12:00. Captured birds were identified to species using a field guide [25], and aged and sexed when possible using ‘The Australian Bird Bander’s Manual’ [26] and ‘The Bander’s Aid’ [27]. Each bird was fitted with a metal band issued by the ABBBS (Australian Bird and Bat Banding Authority) and standard morphological measurements were taken.

Birds were classified into one of four foraging groups based on previous diet studies [28, 29]: carnivores, insectivores, nectarivore-insectivores, and omnivores. Foraging groups had the following dietary compositions: carnivores = arthropods and vertebrates; insectivores = arthropods; nectarivore-insectivores = arthropods and nectar; and omnivores = seeds, nectar, fruit, arthropods, and vertebrates. Foraging group classification for each bird species caught is provided in the Supplementary Materials (Table A in the S2 Appendix).

### Collection and preservation of samples for isotopic analysis

Blood was sampled (20–100 μl) from the antebrachial vein of each bird using a needle (23–27 gauge, depending on bird body size) and a heparinized capillary tube. Blood samples were transferred from the heparinized capillary tube to a glass slide, allowed to dry, and sealed with another glass slide for preservation until preparation for stable isotope analysis. Stainless steel scissors were used to cut 1–2 mm from the tips of four claws of each bird. Claw samples were placed in plastic Eppendorf tubes for preservation until preparation for stable isotope analysis. Birds were released following blood and claw tissue sampling and were not re-sampled if caught again during the same two to four day sampling trip.

Potential resource and prey items (basal food sources) for birds were collected during bird sampling periods at each mist-netting site. Plant, arthropod, and vertebrate food sources were collected in the mangrove forests where birds were sampled, and in adjacent woodlands (*Eucalyptus* and *Melaleuca* spp.) and saltmarshes (see Table A in the S3 Appendix for a full description of the basal food sources collected and their isotopic values). It should be noted that, while many birds caught may feed on nectar or fruit, these sources were not sampled directly. Instead, given their status as primary producers, leaf samples were collected with the assumption that they should have similar isotopic values to nectar and fruit. Comparison of isotopic values among different anatomical plant parts has shown that variation is generally small (between 2 and 3% for δ^15^N; [30]). Furthermore, Codron et al. 2005 [31] did not find substantial differences in the δ^15^N and δ^13^C values of leaves, flowers, and fruit in South African savannah trees. However, because anatomical differences in isotopic values have not been explicitly tested for in the present study, the assumption that leaves have similar isotopic values to nectar and fruit will be taken into consideration when interpreting results.

Plant matter and crabs were collected by hand, insects by beating and direct searching [32], and fish by cast-net. Once collected, all basal food sources were immediately placed on ice, and subsequently stored frozen until preparation for stable isotope analysis.

### Sample preparation and isotopic analysis

Five leaf samples were pooled for each species of plant in preparation for isotopic analysis. White muscle tissue was excised from chelae and legs of crabs, and from below the dorsal fin in fish. Multiple individuals from each Order or Family of insects were pooled and left whole for isotopic analysis. Subsequently, all samples were washed in distilled water, oven-dried at 60°C, and homogenized using a bead mill. Claw samples were washed in a 2:1 chloroform:methanol mixture for fifteen minutes with a magnetic stirrer, allowed to air dry for 48 hours under a fume hood, and left un-homogenized for isotopic analysis [33]. Dried blood samples were powdered and homogenized using a metal scraper on the glass slide where samples had been smeared during collection [34].

Samples of blood, claws, and basal food sources were weighed into tin capsules, and carbon and nitrogen stable isotope ratios were determined using a PDZ Europa ANCA-GSL elemental analyzer connected to a PDZ Europa 20–20 isotope ratio mass spectrometer at the University of California Davis Stable Isotope Facility, USA. All stable isotope ratios were expressed in per mil (‰) using the δ notation:

$$\delta X\;=\;\left(\frac{R(sample)}{R(standard)}-1\right)\;\times \;1,000$$

where *X* refers to the element of interest (i.e. C or N) and R is the ratio of the heavier isotope to the lighter isotope of element *X* (i.e. ^13^C/^12^C or ^15^N/^14^N). The δ values are presented relative to the international standards VPDB (Vienna PeeDee Belemnite) for δ^13^C, and Air for δ^15^N.

It should be noted that lipids were not extracted from the baseline resources. The C:N ratios for all animal sources (fish, crabs, and insects) and bird consumers were relatively low (4.09 ± 0.89 and 3.3 ± 0.07, respectively) and therefore it is unlikely that lipid extraction or normalization would influence their δ^13^C values [35]. Also, none of the plant sources had carbon concentrations greater than 40%, and so normalisation for lipid content was not necessary [35].

### Data analysis

#### Foraging group identification

There are many clustering techniques (e.g. hierarchical, partitioning, etc.) available for determining statistical groups that occur within multivariate data. When prior biological knowledge is available to inform statistical clustering of data, two indexes have been developed to validate cluster algorithm choice and the number of clusters in the data: 1) the biological homogeneity index (BHI) and 2) the biological stability index (BSI) [36]. Biological validation using BHI and BSI quantify the ability of unsupervised clustering algorithms to provide biologically meaningful clusters [36].

Given the average isotopic signature of blood tissues for each bird species, and prior knowledge of their placement in foraging groups identified from previous diet studies, the R package *clValid* was used to choose the best clustering algorithm (‘hierarchical’, ‘k-means clustering’, or ‘partitioning around medoids’) and to identify the number of clusters in the data (ranging from 2 to 8; [37]). The biological validation procedure found the ‘partitioning around medoids’ (PAM) clustering algorithm and three clusters to provide the most biologically meaningful grouping of bird blood isotopic values at both sites. Subsequently, the PAM clustering algorithm with the Manhattan distance metric was used to group species’ average blood δ^13^C and δ^15^N values into three clusters at both Cocoa Creek and Healy Creek, separately [38, 39]. The R package *factoextra* was used to visualise cluster analysis results at both sites [40].

#### Isotopic turnover rate and application of stable isotope discrimination factors

Isotopic turnover rates measure the time required for animal tissues integrate resource isotopic values, and tissue discrimination factors measure the difference between resource and tissue discrimination factors that arise due to physiological processes (i.e. excretion and assimilation). Avian blood and claw tissues differ in their isotopic turnover rates and discrimination factors [21, 22]. Due to differences in turnover rate, whole blood integrates food source isotopic ratios from the previous 2–3 weeks [41] and claw tissue (specifically the distal 1-2mm ‘tip’ of the claw) integrates isotopic ratios from the previous 2–3 months [42]. Therefore, isotopic values in consumer blood and claw tissues can provide a temporal comparison of individual niche size and overlap.

The majority of birds caught in the study were of the Order Passeriformes. Discrimination factors (Δ^13^C and Δ^15^N) for passerine blood and feather tissue have been measured in multiple studies [43, 44, 45, 46], but there are few studies quantifying passerine claw discrimination factors [33] (but see [22]). Given that claws and feathers are both primarily composed of keratin, and that previous studies have found high correlation between their δ^13^C and δ^15^N values, passerine feather discrimination factors were used for claw tissue in the present study [33].

As well as varying among tissue types, discrimination factors are likely to differ between species and foraging groups with different dietary compositions [41, 44]. However, when investigating a bird assemblage comprised of 31 species, it is not feasible to use species-specific discrimination factors. Instead, we have chosen to use discrimination factors that have been determined for multiple foraging groups within the Order Passeriformes. We have followed the approach of Ferger et al. (2013) [33] and taken an average of discrimination factors, separately for blood and feathers, across multiple passerine foraging groups (omnivores, insectivores, frugivores, and granivores). Using average discrimination factors can provide misleading results if they deviate significantly from species-specific consumer-prey discrimination factors [47, 48]. However, the main objective of the present study was to compare the relative isotopic niche size, overlap, degree of individual specialisation, and source contribution among foraging groups occurring within sites. Therefore, average discrimination factors may not be entirely accurate for individual bird species, but comparisons among foraging groups should be robust.

Following averaging across passerine foraging groups, discrimination factors were calculated as Δ^13^C = 1.81 ± 1.45 and Δ^15^N = 2.23 ± 0.39 for blood tissue, and Δ^13^C = 3.04 ± 0.9 and Δ^15^N = 3.42 ± 0.36 for claw tissue. Discrimination factors were re-calculated as averages of foraging group mean discrimination factors determined by studies that used experimental feeding trials (collated in the Supplementary Materials of Ferger et al. 2013 [33]). To directly compare isotopic niche size and overlap in blood and claw tissues, bird isotopic values were reconstituted by subtracting the Δ^13^C and Δ^15^N value specific to each tissue from raw δ^13^C and δ^15^N values. Discrimination factors for blood tissue were also used in stable isotope mixing models to allow bird δ^13^C and δ^15^N values to be related to basal food source isotopic values, and the standard deviation associated with each discrimination factor was used to incorporate natural variability into estimates of dietary composition (see ‘Relative contribution of sources to foraging group diet’ subsection below for further explanation).

#### Isotopic niche size and overlap

Boxplots of foraging group blood and claw δ^13^C and δ^15^N values at each sampling site were inspected, and extreme outliers outside of the expected range were removed. It is possible that these outliers represented individual specialists, however they were not considered representative of the entire foraging group, and their removal was necessary to meet assumptions of multivariate normality. Multivariate normality in δ^13^C and δ^15^N values is an assumption of Bayesian isotopic niche size and overlap calculations [16, 18], and was assessed for using the function ‘mshapiro.test’ in the R package *RVAideMemoire* [49]. Additionally, differences in bird blood and claw δ^13^C and δ^15^N values across seasons and sites were assessed using Welch’s two-sample t-tests (Table A and C in the S4 Appendix).

Bird reconstituted δ^13^C and δ^15^N values were used to determine the isotopic niche size and overlap of bird foraging groups identified from cluster analysis. Isotopic niche size was calculated using standard Bayesian ellipse areas (hereafter referred to as ‘ellipse areas’ or ‘SEA_B_’) in the R package *SIBER*, which provides an estimate of each foraging group’s dietary variability and indicates their degree of generalism or specialism [16]. Subsequently, pairwise comparisons of tissue and foraging group SEA_B_’s determined the probability that one group’s posterior distribution was smaller or larger than another [16]. Finally, the probability of isotopic niche overlap among foraging groups was calculated in the R package *nicheROVER* [50].

Due to variability in baseline resource isotopic values between sites, it is often necessary to standardize measures of isotopic niche size and overlap when making direct site comparisons [15, 51, 52]. However, we were primarily interested in within-site comparisons of isotopic niche size and overlap among foraging groups, rather than in making direct site comparisons. Additionally, due to the capture of different species at each site, direct comparison of foraging group niche sizes between sites was not prudent. Therefore, we chose to forego site standardization, and readers should be cautious about making any between-site comparison of isotopic niche size and overlap in this study.

#### Individual dietary specialisation

To further resolve issues in defining generalist vs. specialist outlined in Fig A in S1 Appendix, we quantified the degree of individual dietary specialization within each foraging group. The raw isotope values of individual consumers for which both blood and claw samples had been collected were used to calculate the relative index of specialization (RIS) for each foraging group [53, 54], and calculations were performed separately for carbon and nitrogen isotope values. Calculating RIS separately for δ^13^C and δ^15^N values was performed to provide an indication of individual specialisation across habitat (δ^13^C) or trophic level (δ^15^N) for each foraging group. More specifically, analysis of variance (ANOVA) was used to evaluate variability in isotope values both within and between individuals in each foraging group, i.e. δ^13^C or δ^15^N values were a function of tissue type (blood vs. claw) and ‘individual’ was a random factor [54]. The residual mean sum of squares was used to represent variability within individuals (WIC, within-individual component) and the mean sum of squares of the ‘individual’ random factor was used to represent variability between individuals (BIC) [54]. RIS was then calculated as BIC/WIC, providing a measure of the degree of specialization that is robust to temporal variation and comparable across species and foraging groups [53, 54] (See Table A in S8 Appendix for WIS, BIC, and RIS values). RIS can be artificially depressed when the distribution of isotope values is bimodal [54], and histograms of δ^13^C and δ^15^N values for each foraging group were visually inspected prior to analysis.

#### Relative contribution of sources to foraging group diet

For foraging group food web construction, Bayesian stable isotope mixing models were used to determine the probable relative contribution of basal food sources to the diet of mangrove bird isotope-based foraging groups at each sampling site. Mixing models require differentiation in source isotopic values to reach a solution, and their discriminatory power decreases as the number of sources increases [17]. Therefore, sources that are not significantly distinct in their isotopic values can be grouped prior to running mixing models [17]. Biological interpretability should also be considered when grouping sources *a priori* [17, 55]. Therefore, in the present study, sources that had overlapping error bars (± sd) and were biologically similar were grouped as follows: mangrove primary (M), woodland primary (W), mangrove fish (F), mangrove crab (C), forest insect (I), and saltmarsh crab and insect (S) (see Table 1 for description).

**Table 1:** A description of the basal resources grouped into sources for use in stable isotope mixing models.

Source	Description of source isotopic values*
Mangrove primary (M)	Primary production (leaves) to represent nectar and fruit resources from mangroves
Woodland primary (W)	Primary production (leaves) to represent nectar and fruit resources from terrestrial woodlands and forests (*Eucalyptus* and *Melaleuca* spp.)
Saltmarsh crab and insect (S)	Insect and crab prey from saltmarsh
Forest Insect (I)	Insect prey from all forest types (mangrove and terrestrial)
Mangrove crab (C)	Crab prey from mangroves
Mangrove fish (F)	Fish prey from mangroves (includes estuarine fish species and mudskippers)

*Detailed identification of all basal food resources collected and their isotopic values can be found in the Supplementary Materials (Table A in the S3 Appendix).

Mixing models were run separately for each foraging group at each sampling site, and only basal food sources that are known to be consumed by birds in each foraging group were used (through consultation of previous dietary studies). Also, because basal food sources were collected when birds were sampled, only bird blood δ^13^C and δ^15^N isotopic values were used in the mixing models because blood tissue most closely reflects diet integration at that time.

Prior to running the mixing models, mixing biplots were used to satisfy the assumption that all consumers lie within source polygons [56]. Three individual consumers lay outside of the 95% mixing region (Fig A in the S5 Appendix), and were removed prior to running the mixing models in the R package *simmr* [57]. Bayesian stable isotope mixing models allow variability in discrimination factors and resource concentration dependence values to be incorporated and provide a measure of how uncertain estimates of relative source contributions are in the form of posterior probability distributions [17]. To meet the assumption of complete mixing, model convergence was confirmed using Gelman convergence diagnostics [58]. Although we grouped sources *a priori* to obtain a manageable number of sources, some source pairs were still unable to be distinguished by mixing models due to similarity in their isotopic values. Therefore, following protocol outlined in Phillips et al. (2014) [17], an *a posteriori* approach was used to further combine sources that had high negative correlation.

All statistical analyses were performed in R version 3.3.2 [59].

## Results

A total of 31 bird species comprising five literature-based foraging groups were caught during wet and dry seasons across both mangrove sampling sites (Table A in the S2 Appendix and Fig A in the S6 Appendix). Only two individuals of one granivorous bird species (*Geopelia striata*) were caught, and therefore the granivore foraging group was removed from subsequent analyses. There was no difference in blood or claw isotopic values across wet and dry season sampling periods at Cocoa Creek, except for δ^15^N values in claw tissue that were higher on average in the dry season by 0.5‰ (*t* = -3.25, df = 111.95, p = 0.002; Table A in the S4 Appendix). However, because the trophic fractionation of nitrogen stable isotopes is known to be 3–5‰ [60], a 0.5‰ difference between seasons was not large enough to warrant separate analyses. Therefore, isotopic values were pooled across seasons at Cocoa Creek, separately for blood and claw tissues, in all subsequent analyses. As noted above, birds were only sampled from Healy Creek during the wet season.

### Foraging group identification

**Fig 2:**
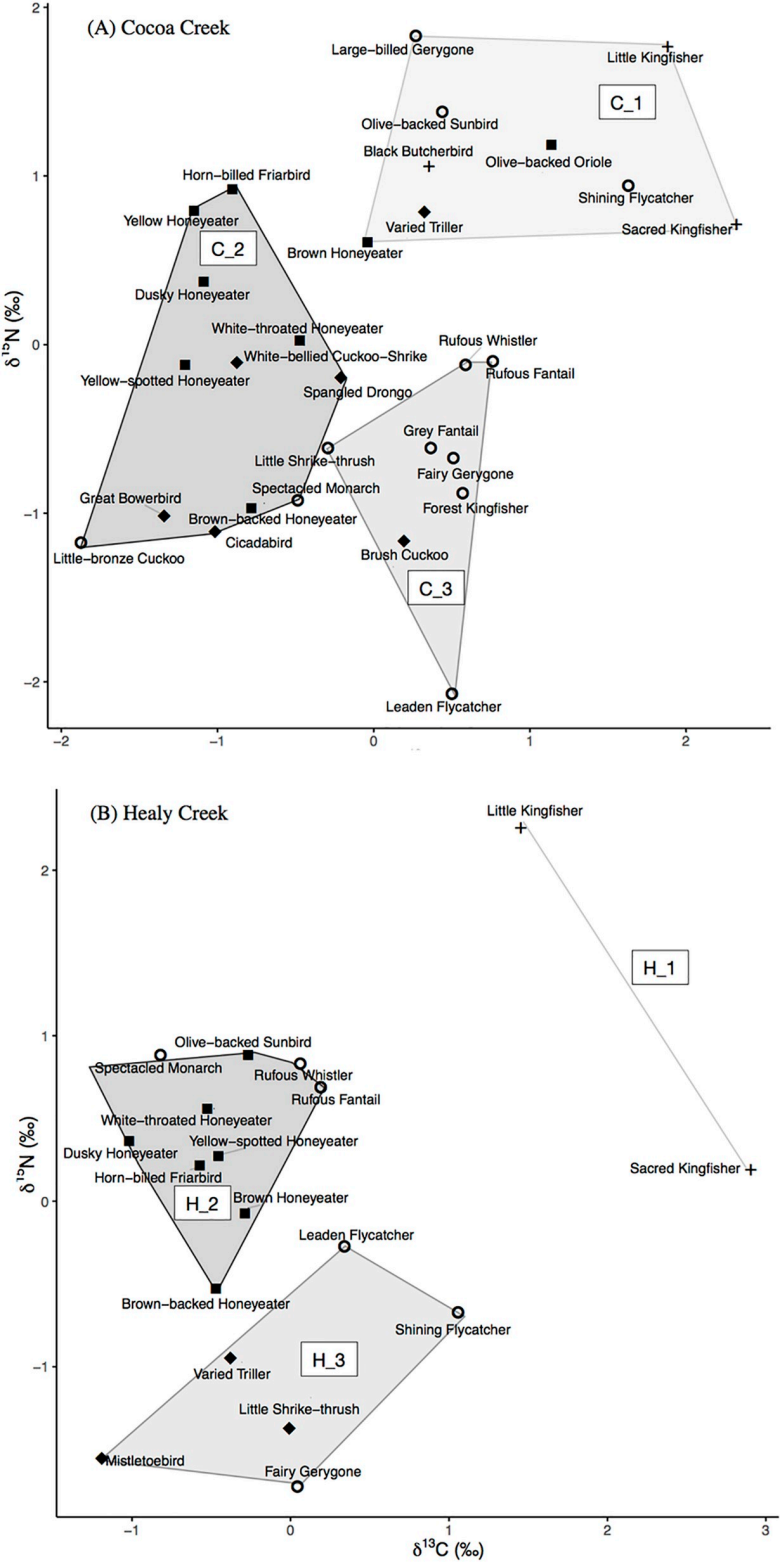
Cluster analysis showing bird species grouped by their blood δ^13^C and δ^15^N values at mangrove forest sites. (A) Cocoa Creek and (B) Healy Creek. Symbols indicate each species’ foraging group as defined by the literature: omnivore (diamond), nectarivore-insectivore (square), carnivore (plus sign), and insectivore (open circle). Convex hulls delineate the three isotope-based foraging groups identified by cluster analysis at Cocoa Creek (C_1, C_2, C_3) and Healy Creek (H_1, H_2, H_3). The axes show standardized values of bird blood δ^13^C and δ^15^N values (mean = 0, standard deviation = 1).

Cluster analysis demonstrated that blood δ^13^C and δ^15^N values of mangrove bird species did not fully correspond to their foraging group classification by previous diet studies (i.e. carnivores, insectivores, nectarivore-insectivores, and omnivores; Fig 2). Additionally, grouping of bird species by their isotopic values was not the same at the two mangrove sites. At Cocoa Creek, two isotope groups were comprised of a mix of omnivorous, carnivorous, nectarivorous-insectivorous, and insectivorous bird species (C_1, C_2; Fig 2A). However, the third isotope group (C_3) had less dietary diversity among its bird species, consisting mainly of insectivorous bird species and one omnivore (Fig 2A). At Healy Creek, carnivorous Sacred Kingfishers (*Todiramphus sanctus*) and Little Kingfishers (*Ceyx pusillus*) were grouped separately from all other bird species (H_1, Fig 2B). The second isotope group at Healy Creek was comprised of insectivorous and nectarivorous-insectivorous bird species (H_2, Fig 2B), while the third isotope group was comprised of omnivorous and insectivorous bird species (H_3, Fig 2B).

Isotope-based foraging groups identified from cluster analysis were used in all subsequent analyses of mangrove bird foraging group isotopic niche size, overlap, individual specialisation, and source contribution at Cocoa Creek and Healy Creek.

### Isotopic niche size and overlap

**Fig 3:**
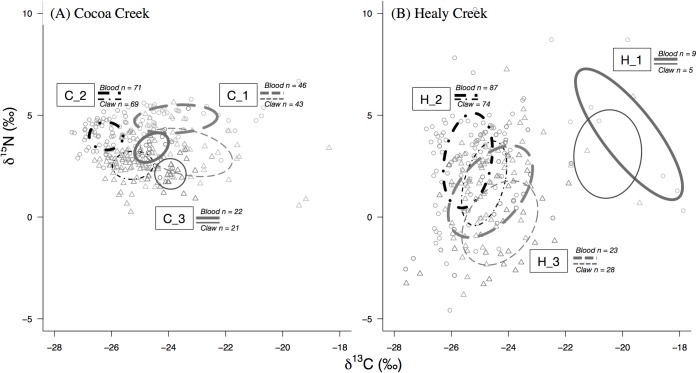
Standard Bayesian ellipse areas (SEA_B_) represent the isotopic niches of mangrove bird isotope-based foraging groups at two mangrove sampling sites. (A) Cocoa Creek and (B) Healy Creek. Line colour and type differentiate between isotope-based foraging groups, and line thickness indicates tissue type (thicker-lined ellipses show blood isotopic niches, and thinner-lined ellipses show claw isotopic niches for each group). Individual consumer isotopic values are also displayed (circles = blood, triangles = claw). Note: between sites the isotope-based foraging groups are comprised of different species and therefore should not be directly compared.

Isotopic niche measures dietary trophic diversity, and can therefore provide an indication of how generalised or specialised the collective diet of individual foraging groups are [14, 19]. The overlap of foraging group isotopic niches determines dietary similarity among groups, and therefore the degree to which individuals might be competing for resources [18]. Isotopic niches were measured by calculation of standard Bayesian ellipse areas (SEA_B_) using reconstituted blood and claw isotopic values for isotope-based foraging groups (Fig 3). SEA_B_ calculation is not robust under when sample sizes are < 10 [16], and it should be noted that isotope group H_1 had sample sizes of 9 and 5 for blood and claw tissues, respectively. Therefore, it is possible that due to small sample size the ellipse areas of H_1’s blood and claw tissues are underestimated [16].

Reconstituted claw δ^15^N values were generally lower than reconstituted blood δ^15^N values, with an average difference across foraging groups of 1.45 ‰ ± 0.09 standard deviations and 1.64 ‰ ± 0.84 standard deviations at Cocoa Creek and Healy Creek, respectively (Fig 3). The consistently low values of reconstituted claw δ^15^N values across foraging groups and sites suggest that this pattern is likely an artefact of the trophic discrimination factors used. Furthermore, this consistency indicates that discrimination did not differ among forging groups, and that our use of average discrimination factors to make comparisons of relative isotopic niche size, overlap, and source contributions among foraging groups should be robust. However, differences between reconstituted blood and claw isotopic values could also be associated with temporal variability in resource isotopic values and by the movement of individuals between habitats.

The ranges of δ^15^N isotopic values in blood and claw tissues were larger at Healy Creek than at Cocoa Creek, which corresponds to a larger range in baseline source δ^15^N isotopic values at Healy Creek (all δ^15^N range differences between sites in blood, claw, and baseline isotopic source values were significant at p<0.05, Table C and D in the S4 Appendix). However, due to lack of site-standardization, ellipse areas cannot be directly compared between sites. Therefore, the following subsections make only within-site comparisons of isotopic niche size and probability of niche overlap. For all probabilistic pairwise comparisons of isotopic niche size (described below), see Table A and B in the S7 Appendix.

### Tissue comparisons (i.e. short-term vs. long-term isotopic integration)

**Fig 4:**
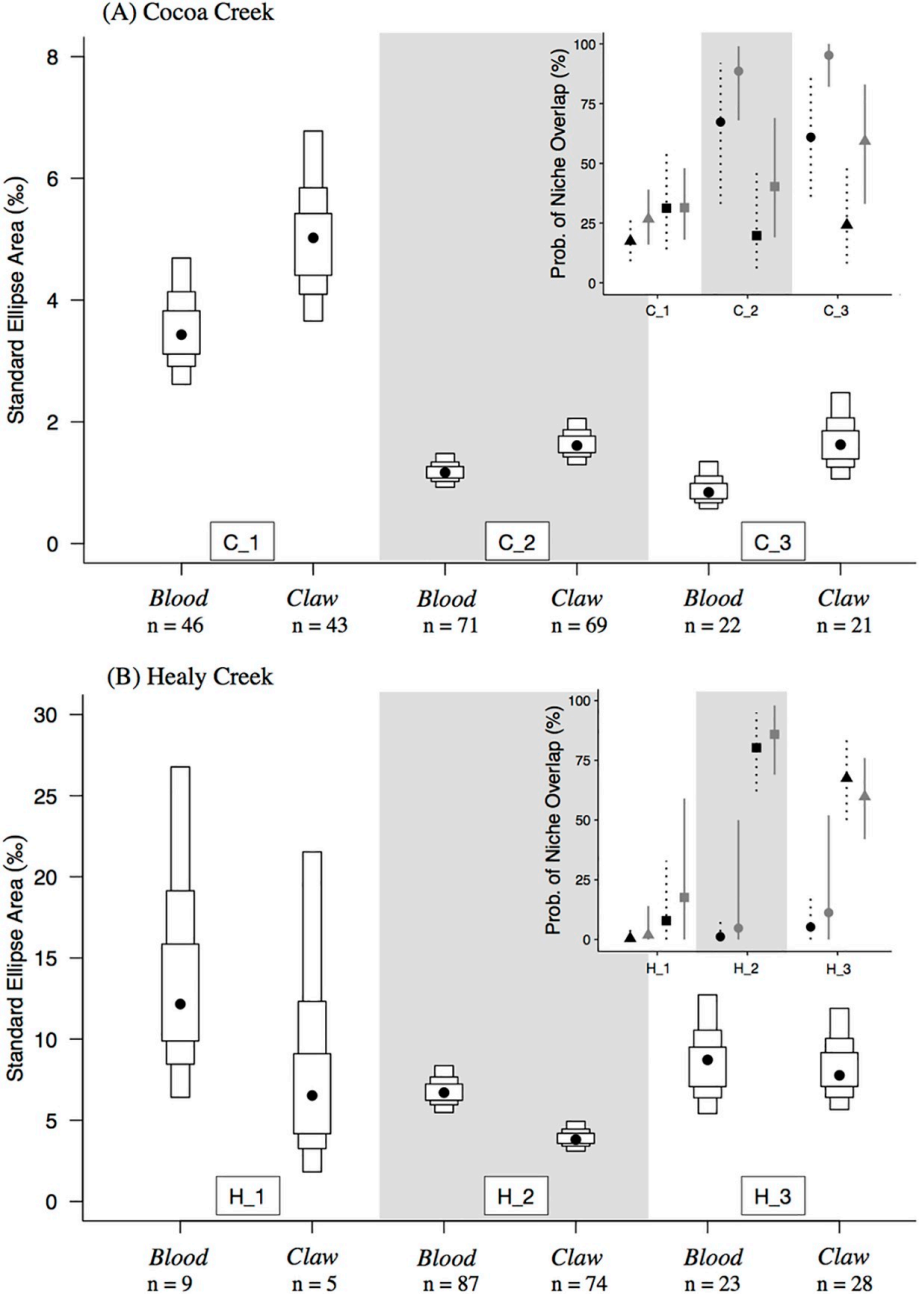
Standard Bayesian ellipse areas (SEA_B_) for blood and claw tissues of mangrove bird isotope-based foraging groups. (A) Cocoa Creek and (B) Healy Creek. Black circles are the mode SEA_B_, and boxes show the 50%, 75%, and 95% credible intervals. Inset plots show the probability of isotopic niche overlap (mean ± 95% credible intervals) among the foraging groups. In the inset plots, colour indicates tissue type (‘black’ = blood, ‘grey’ = claw), and symbols represent the foraging groups as follows: (circle) = C_1 or H_1; (triangle) = C_2 or H_2; (square) = C_3 or H_3.

Given that blood and claw integrate isotopic values of resources consumed over different time frames (short-term vs. long-term), comparison of their isotopic values can indicate whether resources consumed by each foraging group is seasonally consistent. Within each site, ellipse areas differed between blood and claw tissues of some foraging groups, suggesting temporal changes in their foraging strategies (Fig 4). At Cocoa Creek, all isotope groups had larger claw ellipse areas in comparison to blood (probability claw > blood ranged between 95–98% for C_1, C_2, and C_3; Fig 4A). Probability of isotopic niche overlap (Pr(INO)) between isotope groups C_1 and C_2 was similar in claw and blood tissues, while C_3 had lower Pr(INO) in blood tissue compared to claw tissue (Fig 4A). At Healy Creek, ellipse areas of blood and claw tissues were similar in most isotope groups except for H_2, which had larger blood ellipse areas in comparison to claw ellipse areas (probability blood > claw = 100%, Fig 4B). Also, Pr(INO) did not differ between blood and claw tissues for any of the isotope foraging groups at Healy Creek (Fig 4B).

### Foraging group comparisons

At Cocoa Creek, C_1 had the largest ellipse areas in comparison to other isotope foraging groups (probability C_1 > C_2 or C_3 = 100%) and had low to intermediate Pr(INO) with other foraging groups (Fig 4A). Isotope group C_2 had a larger blood ellipse areas than isotope group C_3 (probability C_2 > C_3 = 87%), and these two foraging groups had low to intermediate Pr(INO) with each other (Fig 4A). At Healy Creek, H_1 had the largest blood ellipse areas (probability H_1 > H_2 or H_3 = 99% and 91%, respectively) and low Pr(INO) with other isotope foraging groups (Fig 4B). Isotope groups H_2 and H_3 had similar ellipse areas except for H_2’s claw ellipse area, which was smaller than all other isotope groups (probability H_2 < H_1 or H_3 = 93% and 100%, respectively; Fig 4B). Also, isotope groups H_2 and H_3 had intermediate to high Pr(INO) with each other (Fig 4B). (Note that H_1 had small relatively sample sizes of 9 and 5 for blood and claws, respectively, increasing the possibility that their ellipse areas have been underestimated [16].)

### Individual dietary specialisation

**Fig 5:**
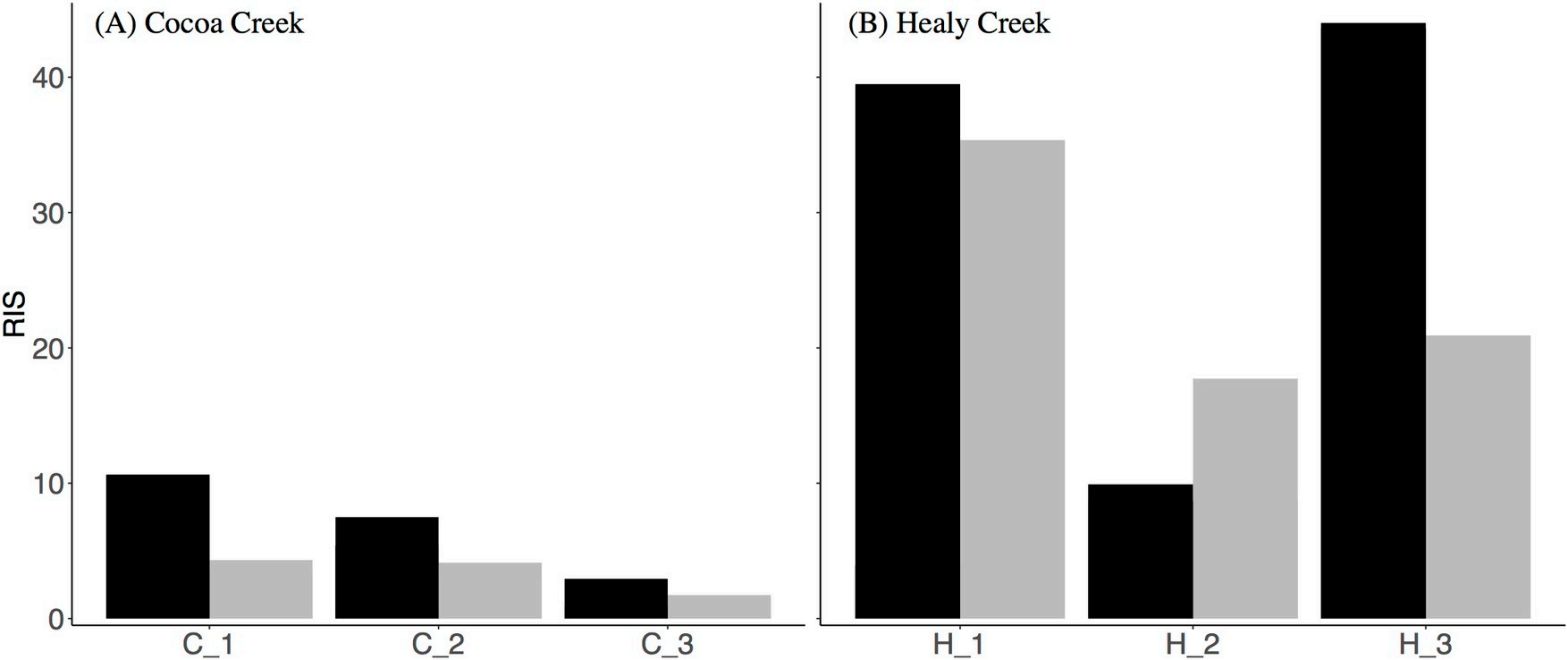
Relative index of specialisation (RIS) for δ^13^C and δ^15^N values of mangrove-bird isotope-based foraging groups. (A) Cocoa Creek and (B) Healy Creek. Black bars = δ^13^C; Grey bars = δ^15^N.

Overall, the degree of individual foraging specialization in isotope foraging groups was higher at Healy Creek in comparison to Cocoa Creek for both δ^13^C and δ^15^N values, and generally higher for δ^13^C values than for δ^15^N values (except for H_2; Fig 5). At Cocoa Creek, C_2 had the highest RIS values (Fig 5A), corresponding with its high dietary diversity (i.e. C_2 is comprised of carnivores, omnivores, insectivores, and nectarivore-insectivores). Alternatively, RIS values did not correspond with dietary diversity at Healy Creek, with carnivores in H_1 having the highest RIS value (Fig 5B).

Given limitations associated with small sample sizes and lack of complete sampling replication across seasons and sites, all isotopic niche size, overlap, and individual specialization analyses were performed separately for three individual species chosen to validate broader conclusions for isotopic foraging groups. Individual species chosen for separate analyses had sample sizes greater than 10 (except for the Sacred Kingfisher at Healy Creek) and represent different movement categories: Dusky Honeyeater (sedentary and nomadic), Olive-backed Sunbird (sedentary), and Sacred Kingfisher (partially migratory) (see S9 Appendix for results). Overall, results confirm the broader findings that species’ foraging strategies, particularly individual dietary specialisation (Fig A in S9 Appendix), differ between sampling locations.

### Relative contribution of sources to foraging group diet

**Fig 6:**
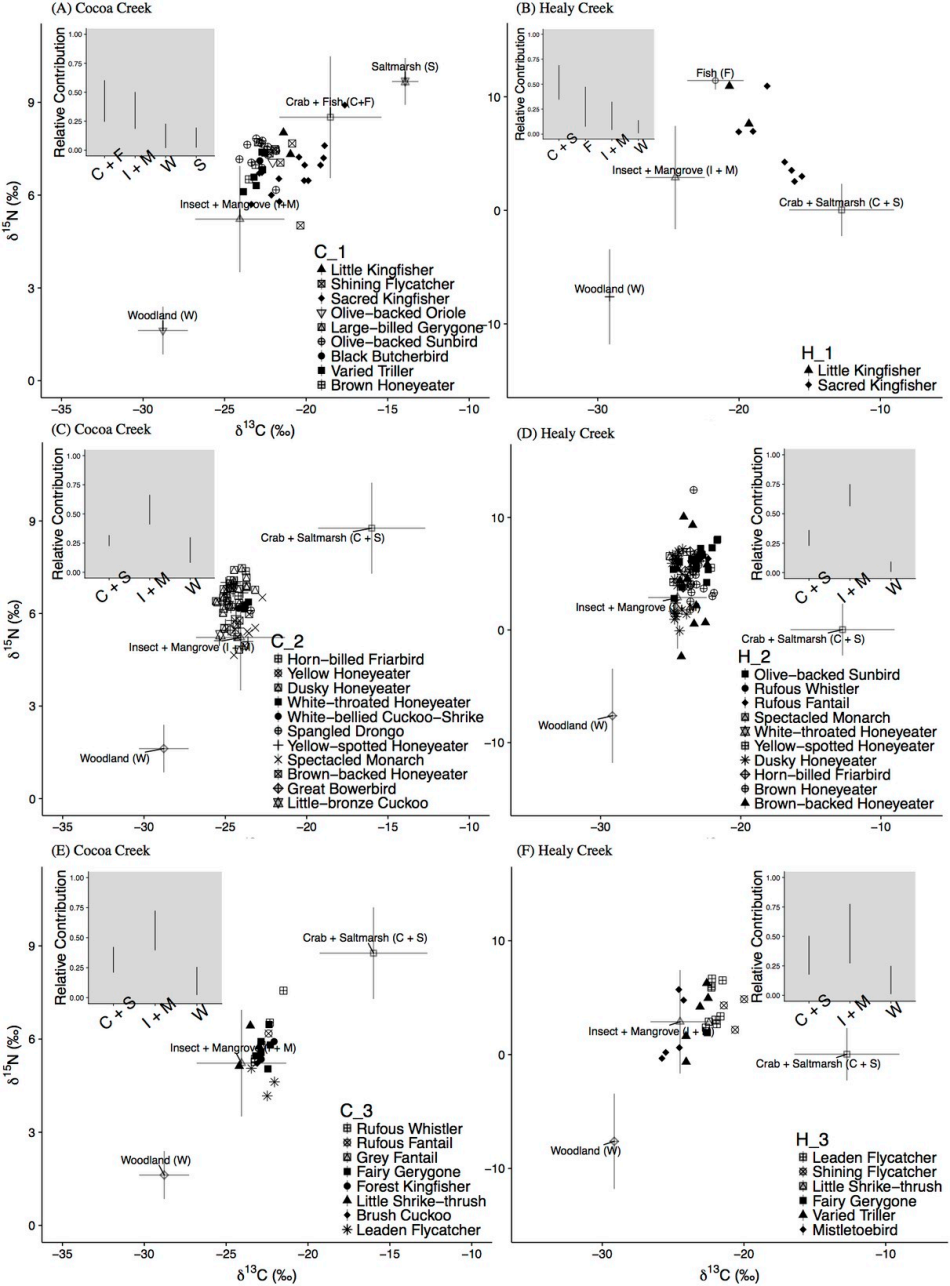
δ^13^C and δ^15^N values of sources (mean ± sd) and mangrove bird consumers at Cocoa Creek and Healy Creek for each isotope-based foraging group. Sources that were unable to be distinguished by mixing models were combined. Inset plots show the probable relative contribution of sources (95% credible interval ranges), as estimated by mixing models, to the diet of each foraging group.

Bayesian stable isotope mixing models were used to determine the probable relative contribution of coastal basal resources to the diet of mangrove bird isotope-based foraging groups (Fig 6). However, given that mixing models for all foraging groups were underdetermined (i.e. too many sources and not enough isotopic tracers), it was not prudent to evaluate mean relative contribution values because unique solutions were not possible [61, 62]. Therefore, the relative likelihoods of source contributions for each foraging group were reported as 95% credible interval ranges (Fig 6), and caution was exercised in their interpretation so that all feasible solutions were considered.

Prior to running mixing model analyses, simulated mixing regions for consumer-source biplots validated mixing models for all isotope foraging groups at both sites. Following *a posteriori* combination of sources that could not be distinguished by mixing model analyses [17], mixing models demonstrated that the relative contribution of sources to isotope foraging groups differed between sites, and was dependent on their species composition (Fig 6). At Cocoa Creek, isotope group C_1 was comprised of a mix of carnivorous, insectivorous, omnivorous, and nectarivorous-insectivorous species, and their collective diet appeared to be primarily supported by crab, fish, insect, and mangrove primary sources (Fig 6A). Alternatively, isotope group H_1 at Healy Creek was comprised of only carnivorous bird species, and their collective diet appeared to be primarily supported by crab and saltmarsh sources (Fig 6B).

At Cocoa Creek, isotope group C_2 was composed of insectivorous, omnivorous, and nectarivorous-insectivorous species that likely foraged mainly on insect and mangrove primary sources, and secondarily on crab and saltmarsh sources (Fig 6C). Similarly, isotope group H_2 at Healy Creek was composed of nectarivorous-insectivorous and insectivorous species whose diet appeared to be supported mainly by insect and mangrove primary sources, and relatively less crab and saltmarsh sources (Fig 6D).

At Cocoa Creek, isotope group C_3 consisted of primarily insectivorous bird species and one omnivorous species, and their collective diet appeared to consist primarily of insect, mangrove primary, crab, and saltmarsh sources (Fig 6E). Likewise, at Healy Creek, isotope group H_3 consisted of insectivorous and omnivorous species whose diet appeared to be supported primarily by insect, mangrove primary, crab, and saltmarsh sources (Fig 6F).

## Discussion

Environmental heterogeneity in resource availability can cause opportunistic and generalised foraging by resident species [1, 63, 64]. The present study has provided insight into the foraging strategies and food webs of birds inhabiting a complex and dynamic coastal mangrove environment. The isotopic values of mangrove bird assemblages suggest that bird foraging strategies are more opportunistic and generalised than expected by previous diet studies (collated in [28, 29]).

### Isotopic insights to mangrove bird foraging ecology

Two sites were used in the present study to determine if bird foraging patterns were spatially consistent. The baseline δ^13^C ranges were similar at both mangrove sites, however Healy Creek had depleted δ^15^N source values resulting in a larger baseline δ^15^N range. Despite differences in δ^15^N range, carnivorous, nectarivorous-insectivorous, and insectivorous bird species occupied similar positions relative to sources in isotope spaces at Cocoa Creek and Healy Creek. However, between sites, there were differences in the organization of bird species into isotopic foraging groups and their respective foraging strategies.

### Correspondence between literature- and isotope-based foraging groups

The organization of mangrove forest bird species into isotope-based foraging groups implied opportunistic resource use, as isotopic foraging clusters did not strictly correspond to bird species’ foraging group classification by previous diet studies (i.e. their observed diet). The only isotope group that did correspond to literature-based classification was a Healy Creek cluster that was comprised of two carnivorous bird species (i.e. H_1), and they had higher isotopic separation from each other relative to species within other clusters. The overall prevalence of opportunism in mangrove forest birds corroborates previous research of their foraging ecology, which has been well studied in the Northern Territory of Australia using visual observation to describe a bird assemblage that is dominated by opportunistic insect foragers [12, 65]. However, until now, similar research has been limited for mangrove bird assemblages of northeastern Australia, which contain a higher abundance of facultative bird species that are known to forage in adjacent coastal woodlands and wetlands seasonally [66]. Furthermore, the utility of an isotopic approach in this system has not been previously investigated.

### Isotopic niche size and overlap as an indicator of dietary diversity

At Cocoa Creek, the dietary diversity of birds within isotope groups was associated with isotopic niche size and overlap, meaning that isotope groups with greater observed dietary diversity among their species (i.e. carnivorous, omnivorous, insectivorous, or nectarivorous-insectivorous) had larger isotopic niche sizes and higher prevalence of individual dietary specialisation. Isotope group C_1 was comprised of bird species with all four observed diets, had the largest isotopic niche size, and the highest degree of individual specialisation relative to other foraging groups at Cocoa Creek. Notably, carnivores were grouped with other insectivorous and omnivorous bird species in C_1 (e.g. the Shining Flycatcher (*Myiagra alecto*) and Olive-backed Oriole (*Oriolus sagittatus*)), revealing that these species may opportunistically forage on crab and fish sources as well (corroborated by visual observation of Shining Flycatcher crab foraging; C.A. Buelow 2015, pers. comm., June). Alternatively, insects may also support carnivorous diets at Cocoa Creek. In contrast to the observed dietary diversity of C_1, isotope group C_3 was comprised primarily of insectivorous species (with one omnivore), had the smallest isotopic niche size, and had the lowest degree of individual dietary specialisation; all of which are likely congruent with a smaller resource base.

Isotope groups at Cocoa Creek had primarily low to intermediate probability of isotopic niche overlap, which suggests that, as a whole, isotope groups at this site are foraging on different resources from one another. Supporting this suggestion, mixing models show that all three isotope groups forage to some extent on forest insect and mangrove primary sources, and differ by probable relative contribution of crab, fish, and saltmarsh sources to their collective diets. Not surprisingly, mangrove primary and forest insect sources had the highest probable contribution to isotope group C_2 (~ 40–70%), which had a high abundance of nectarivorous and insectivorous bird species.

In comparison to Cocoa Creek, isotope groups at Healy Creek did not show similarly high observed dietary diversity among their constituent species. Also, further in contrast to Cocoa Creek, higher observed dietary diversity was not consistently associated with larger isotopic niche size or high individual specialisation; rather, the opposite was true. Isotope groups H_2 and H_3, each comprised of species with two different diets, had smaller isotopic niche sizes than H_1, which was comprised of carnivorous species only. Isotope groups H_2 and H_3 also had intermediate to high probability of niche overlap with each other, suggesting that species within these groups forage on similar resources (i.e. insect and mangrove sources, as indicated by mixing models).

The lack of a clear observed dietary diversity-isotopic niche size relationship at Healy Creek may indicate that two different specialist foraging strategies occur within isotope groups at this site. For example, isotope group H_1 may be comprised of individual specialists with different preferences for fish, crab, or saltmarsh resources. This is corroborated by a high degree of individual dietary specialisation and relatively similar contribution of fish, crab, and saltmarsh resources to the collective diet of carnivores in isotope group H_1. Alternatively, isotope groups H_2 and H_3 may be comprised of individuals employing specialized foraging strategies with similar resource preferences, as mixing models confirm that these groups appear to be supported primarily by forest insect and mangrove primary sources. However, H_3 has a relatively high degree of individual dietary specialisation, particularly across its δ^13^C niche axis, suggesting that individuals in this group are specialising on prey resources primarily by habitat type.

There are several ecological mechanisms that could underpin the divergent dietary diversity-isotopic niche size relationships observed between sites. Landscape heterogeneity, resource availability, habitat fragmentation, and species interactions (e.g. competition) are all factors that may influence these relationships [67, 68]. Quantifying these factors at each site would further our understanding of the processes shaping the observed patterns, and requires more attention in the future.

### Temporal changes in foraging strategies: tissue comparisons

The claw isotopic niches of all isotope groups at Cocoa Creek were consistently larger relative to their blood isotopic niches, suggesting either temporal opportunism in their foraging strategies or dietary-switching as resource availability and abundance changes seasonally. For example, seasonally shifting foraging strategies may reflect an increase in insect availability during the wet season in mangrove forests. However, at Healy Creek, blood and claw isotopic niche sizes were similar, suggesting a low likelihood of temporal opportunism in foraging groups (except for H_2, which had a relatively larger blood isotopic niche size). These findings are supported by overall patterns of individual dietary specialization at both sites. Individual dietary specialisation was high at Healy Creek, suggesting that individuals forage on the same resources throughout the year. Alternatively, individual dietary specialization was relatively lower at Cocoa Creek suggesting that individuals are more temporally generalized in their foraging strategy. However, because seasonal diet shifting was not consistent between sites, further research is needed to make concrete conclusions regarding seasonal and spatial differences in resource availability for mangrove forest birds in these areas.

### Limitations and recommendations

It should be noted that using isotopic niche size to evaluate generalist vs. specialist foraging strategies could in part be driven by the degree of isotopic distinction between sources. For example, insect resource partitioning by birds may occur at a finer scale than can be examined by stable isotopes. In fact, observational studies have found that mangrove insectivores will select insects by size [12], demonstrating the importance of using a combination of methods (i.e. observational and gut content analyses) when investigating foraging ecology. Future studies should also consider using sulphur stable isotopes to improve isotopic discrimination in coastal environments [69], and have more mangrove sites for comparison. Finally, larger sample sizes of each bird species would allow the evaluation and comparison of species-specific isotopic niche sizes, and better determine the degree to which individual species exhibit specialized vs. generalized foraging strategies.

The differing movement abilities of bird species (i.e. partially migratory, sedentary, or nomadic, Table A in S2 Appendix) is a confounding factor in this study. The blood or claw isotopic signatures of partially migratory or nomadic bird species may not be representative of foraging at the sampling locations in this study if they have moved or migrated across the landscape in the previous weeks or months. A better understanding of the linkages between bird movement and foraging ecology requires tracking or mark-recapture studies, but was outside the scope of this study.

It is important to consider that any solution within a range of possible solutions identified by mixing models (i.e. 95% credible interval ranges) is potentially the real solution [61, 62]. To obtain the most well constrained mixing model contribution estimates, we used prior information to include only sources that are known to be consumed by bird species in each foraging group [70], and our interpretations reflect a combination of the prior information and mixing model outputs. Despite this, our models remained underdetermined and so other solutions in the source-consumer mixing model spaces should not be disregarded. For example, the solution for C_2 (Fig 6C) suggests that Woodland primary sources are unimportant, yet it is possible they could contribute more substantially to C_2’s collective diet than is suggested by the mixing model outputs [61, 62]. Greater confidence in relative source contribution estimates could be garnered by inclusion of more consumer data and the use of additional prior information [70]. However, for the objectives of our study, the mixing model outputs combine with other information to better understand the foraging ecology of mangrove bird assemblages.

### Conclusions

Due to the unpredictably of Australian climate systems and their inter-annual variability, Australian bird species often survive by following resources as they become available and abundant [71]. Most of the bird species in the present study are mangrove-facultative, and are likely to also use adjacent forests and woodlands [66]. Additionally, many species have broad distributions, either along the length of the east coast or across the tropical north of Australia. With large flexibility and range in habitat choice, we would expect the diets of these bird species to differ spatially, and the foraging groups to shift accordingly. Our comparative approach using both isotopic and previously collated traditional diet studies has provided insight to the foraging ecology and food webs of coastal forest bird assemblages, however more research is required to understand the limits to their foraging flexibility to ensure their persistence in Northern Australia.

## Supporting information

S1 Appendix **Fig A. Interpreting isotopic niche size as an indicator of consumer populations having generalised or specialised foraging strategies.** Plots (a) and (b) show hypothetical δ^13^C and δ^15^N isotope space with individuals in generalist consumer populations represented by black dots, and individuals in specialist consumer populations represented by white dots. Four different resources are available to consumers (represented by diamond, triangle, square, and pentagon symbols; with vertical and horizontal lines to indicate variability, and arrows to indicate the relative contributions of each source to generalist populations). Dashed circles or ellipses around consumer populations represent their isotopic niche size. In (a), consumers from a generalist population forage consistently on all four resources, and isotopic averaging results in an isotopic niche that is of similar size to a specialist population foraging on only one resource (square). In (b), some consumers from a generalist population forage more heavily on one resource (diamond), resulting in a larger isotopic niche compared to generalists in (a). Finally, in (b), individual consumers of a specialist population feed separately on two different resources, resulting in a larger isotopic niche relative to specialists in (a). This is not a complete illustration of all isotopic niche size scenarios that are possible, and only provides an indication of the challenges associated with interpretation.
(DOCX)

S2 Appendix **Table A. δ^13^C and δ^15^N values (mean ± sd) and sample size (n) of blood and claw tissues for bird species caught at each sampling site.** Bird species are organised by their literature-based foraging group membership: carnivores, insectivores, nectarivore-insectivores, and omnivores.
(DOCX)

S3 Appendix **Table A. δ^13^C and δ^15^N values (mean ± sd) and sample size (n) of basal food sources at each sampling site during wet and dry seasons.** Sources are grouped by their vegetation type: mangrove, woodland, or saltmarsh.
(DOCX)

S4 Appendix **Table A. Results of Welch’s two-sample t-tests determining differences in bird blood and claw δ^13^C and δ^15^N values between seasons at Cocoa creek.** P-values less than, or equal to, 0.05 are highlighted in bold. **Table B. Results of Welch’s two-sample t-tests determining differences in source δ^13^C and δ^15^N values between seasons at Cocoa creek.** P-values less than, or equal to, 0.05 are highlighted in bold. **Table C. Results of Welch’s two-sample t-tests determining differences in bird blood and claw δ^13^C and δ^15^N values between sites.** P-values less than, or equal to, 0.05 are highlighted in bold. **Table D. Results of Welch’s two-sample t-tests determining differences in source δ^13^C and δ^15^N values between sites.** P-values less than, or equal to, 0.05 are highlighted in bold.
(DOCX)

S5 Appendix **Fig A. Simulated mixing regions for source-consumer biplots, categorized by isotope-based foraging group and sampling site.** Sources are marked by an ‘x’ and consumers are displayed as black dots. The outer-most contour delineates where 5% of the simulated polygons have a solution (i.e. satisfy point-in-polygon) for each consumer (Smith et al. 2013). Consumers outside of the 95% mixing polygons were removed prior to mixing model analysis (i.e. three consumers in Healy creek isotope group 2 (H_2; d).
(DOCX)

S6 Appendix **Fig A. Plots showing (a) abundance of mangrove bird individuals caught by species, (b) abundance of mangrove bird individuals caught in each literature-based foraging group, and (c) richness of mangrove bird species caught in each literature-based foraging group.** Species’ allocation into literature-based foraging groups is shown in Table A2.
(DOCX)

S7 Appendix **Table A. Pairwise comparisons of standard Bayesian ellipse areas (SEA_B_) in blood vs. claw tissues of mangrove bird isotope-based foraging groups at Cocoa creek and Healy creek**. Probabilities that the SEA_B_ of foraging group claw tissue in rows are smaller than foraging group blood tissue in columns are provided. Highlighted in bold are probabilities that are greater than 0.85 or less than 0.15. **Table B. Pairwise comparisons of standard Bayesian ellipse areas (SEA_B_) of mangrove bird isotope-based foraging groups at Cocoa creek and Healy creek in either blood or claw tissues.** Probabilities that the SEA_B_ of foraging groups in rows are smaller than foraging groups in columns are provided. Highlighted in bold are probabilities that are greater than 0.85 or less than 0.15.
(DOCX)

S8 Appendix **Table A. Individual specialisation metrics calculated from δ^13^C and δ^15^N signatures of isotope foraging groups.** N = sample size, TNW = Total niche width, WIC = Within-individual component, BIC = Between-individual component, RIS = Relative index of specialisation.
(DOCX)

S9 Appendix **Fig A. Standard Bayesian ellipse areas (SEA_B_) represent the isotopic niches of mangrove bird species at two mangrove sampling sites.** (A) Cocoa Creek and (B) Healy Creek. Line colour and type differentiate between species, and line thickness indicates tissue type (thicker-lined ellipses show blood isotopic niches, and thinner-lined ellipses show claw isotopic niches for each species). Individual consumer isotopic values are also displayed (circles = blood, triangles = claw). Note: ellipse areas of species with samples sizes less than 10 may be underestimated (i.e. Sacred Kingfisher at Healy Creek). **Fig B. Standard Bayesian ellipse areas (SEA_B_) for blood and claw tissues of individual bird species.** (A) Cocoa Creek and (B) Healy Creek. Black circles are the mode SEA_B_, and boxes show the 50%, 75%, and 95% credible intervals. Inset plots show the probability of isotopic niche overlap (mean ± 95% credible intervals) among the bird species. In the inset plots, colour indicates tissue type (‘black’ = blood, ‘grey’ = claw), and symbols represent each species as follows: (triangle) = Olive-backed Sunbird; (square) = Sacred Kingfisher; (circle) = Dusky Honeyeater. **Fig C. Relative index of specialisation (RIS) for δ^13^C and δ^15^N values of individual bird species.** (A) Cocoa Creek and (B) Healy Creek. Black bars = δ^13^C; Grey bars = δ^15^N.
(DOCX)
