# Supplementary material for: Stable isotopes reveal opportunistic foraging in a spatiotemporally heterogeneous environment: Bird assemblages in mangrove forests
Source: PLoS One. 2018 Nov 15;13(11):e0206145. doi: 10.1371/journal.pone.0206145 (PMC6237324; doi:10.1371/journal.pone.0206145)
Supplement: S1 Appendix — Fig A. Interpreting isotopic niche size as an indicator of consumer populations having generalised or specialised foraging strategies. Plots (a) and (b) show hypothetical δ13C and δ15N isotope space with individuals in generalist consumer populations represented by black dots, and individuals in specialist consumer populations represented by white dots. Four different resources are available to consumers (represented by diamond, triangle, square, and pentagon symbols; with vertical and horizontal lines to indicate variability, and arrows to indicate the relative contributions of each source to generalist populations). Dashed circles or ellipses around consumer populations represent their isotopic niche size. In (a), consumers from a generalist population forage consistently on all four resources, and isotopic averaging results in an isotopic niche that is of similar size to a specialist population foraging on only one resource (square). In (b), some consumers from a generalist population forage more heavily on one resource (diamond), resulting in a larger isotopic niche compared to generalists in (a). Finally, in (b), individual consumers of a specialist population feed separately on two different resources, resulting in a larger isotopic niche relative to specialists in (a). This is not a complete illustration of all isotopic niche size scenarios that are possible, and only provides an indication of the challenges associated with interpretation. (DOCX) [file pone.0206145.s001.docx]

**S1 Appendix**


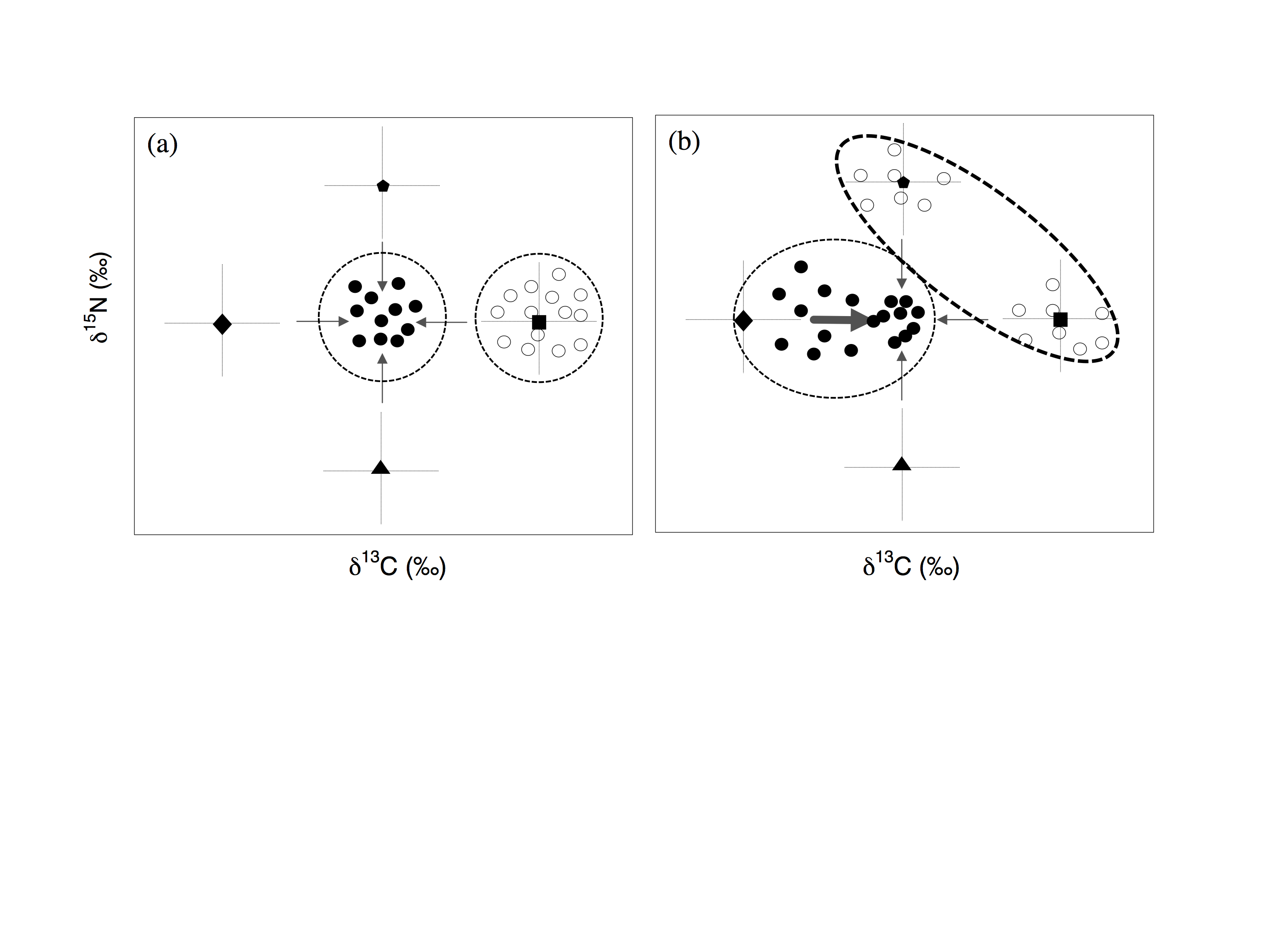


**Fig A.** **Interpreting isotopic niche size as an indicator of consumer populations having generalised or specialised foraging strategies.** Plots (a) and (b) show hypothetical δ^13^C and δ^15^N isotope space with individuals in generalist consumer populations represented by black dots, and individuals in specialist consumer populations represented by white dots. Four different resources are available to consumers (represented by diamond, triangle, square, and pentagon symbols; with vertical and horizontal lines to indicate variability, and arrows to indicate the relative contributions of each source to generalist populations). Dashed circles or ellipses around consumer populations represent their isotopic niche size. In (a), consumers from a generalist population forage consistently on all four resources, and isotopic averaging results in an isotopic niche that is of similar size to a specialist population foraging on only one resource (square). In (b), some consumers from a generalist population forage more heavily on one resource (diamond), resulting in a larger isotopic niche compared to generalists in (a). Finally, in (b), individual consumers of a specialist population feed separately on two different resources, resulting in a larger isotopic niche relative to specialists in (a). This is not a complete illustration of all isotopic niche size scenarios that are possible, and only provides an indication of the challenges associated with interpretation.
