# Supplementary material for: Stable isotopes reveal opportunistic foraging in a spatiotemporally heterogeneous environment: Bird assemblages in mangrove forests
Source: PLoS One. 2018 Nov 15;13(11):e0206145. doi: 10.1371/journal.pone.0206145 (PMC6237324; doi:10.1371/journal.pone.0206145)
Supplement: S2 Appendix — Table A. δ13C and δ15N values (mean ± sd) and sample size (n) of blood and claw tissues for bird species caught at each sampling site. Bird species are organised by their literature-based foraging group membership: carnivores, insectivores, nectarivore-insectivores, and omnivores. (DOCX) [file pone.0206145.s002.docx]

**S2 Appendix**

**Table A. δ^13^C and δ^15^N signatures (mean ± sd) and sample size (n) of blood and claw tissues for bird species caught at each sampling site.** Bird species are organised by their literature-based foraging group membership: carnivores, insectivores, nectarivore-insectivores, and omnivores.

| Bird species | |  | Stable isotope signatures | | | | | |
| --- | --- | --- | --- | --- | --- | --- | --- | --- |
| Common name | Scientific name | Movement | Site | Season | Tissue | n | δ^13^C (‰) ± sd | δ^15^N (‰) ± sd |
| Carnivores | | | | | | | | |
| Black Butcherbird | *Cracticus quoyi* | Sedentary | Cocoa Creek | Winter | Blood | 1 | -22.84 | 7.11 |
|  |  |  |  |  | Claw | 1 | -20.47 | 6.67 |
| Little Kingfisher | *Ceyx pusillus* | Sedentary | Cocoa Creek | Summer | Blood | 1 | -20.96 | 7.32 |
|  |  |  |  |  | Claw | 1 | -22.40 | 6.72 |
|  |  |  |  | Winter | Blood | 1 | -21.39 | 8.02 |
|  |  |  |  |  | Claw | 1 | -20.82 | 9.03 |
|  |  |  | Healy Creek | Winter | Blood | 2 | -19.99 ± 0.98 | 9.28 ± 2.35 |
|  |  |  |  |  | Claw | 2 | -21.24 ± 3.44 | 14.95 ± 12.76 |
| Sacred Kingfisher | *Todiramphus sanctus* | Partially migratory | Cocoa Creek | Summer | Blood | 10 | -20.81 ± 1.99 | 6.83 ± 0.91 |
|  |  |  |  |  | Claw | 8 | -19.38 ± 1.87 | 5.87 ± 0.95 |
|  |  |  |  | Winter | Blood | 7 | -18.45 ± 2.45 | 5.14 ± 2.06 |
|  |  |  |  |  | Claw | 6 | -18.35 ± 1.83 | 5.56 ± 1.25 |
|  |  |  | Healy Creek | Winter | Blood | 4 | -18.45 ± 1.39 | 7.26 ± 2.76 |
|  |  |  |  |  | Claw | 3 | -17.09 ± 1.00 | 6.68 ± 2.62 |
| Insectivores | | | | | | | | |
| Fairy Gerygone | *Gerygone palpebrosa* | Sedentary | Cocoa Creek | Summer | Blood | 5 | -22.57 ± 0.28 | 5.76 ± 0.52 |
|  |  |  |  |  | Claw | 4 | -20.77 ± 0.47 | 4.42 ± 1.17 |
|  |  |  |  | Winter | Blood | 1 | -23.19 | 5.46 |
|  |  |  |  |  | Claw | 4 | -20.9 ± 0.56 | 5.06 ± 0.46 |
|  |  |  | Healy Creek | Winter | Blood | 1 | -22.59 | 1.93 |
|  |  |  |  |  | Claw | 2 | -21.08 ± 0.11 | 1.39 ± 0.45 |
| Forest Kingfisher | *Todiramphus macleayii* | Partially migratory | Cocoa Creek | Summer | Blood | 3 | -22.61 ± 0.47 | 5.55 ± 0.32 |
|  |  |  |  |  | Claw | 2 | -20.85 ± 0.24 | 5.41 ± 0.41 |
| Grey Fantail | *Rhipidura albiscapa* | Partially migratory | Cocoa Creek | Winter | Blood | 1 | -22.84 | 5.81 |
|  |  |  |  |  | Claw | 2 | -21.005 ± 0.90 | 6.81 ± 0.80 |
| Large-billed Gerygone | *Gerygone magnirostris* | Sedentary | Cocoa Creek | Winter | Blood | 1 | -22.95 | 7.71 |
|  |  |  |  |  | Claw | 1 | -20.85 | 7.55 |
|  |  |  | Healy Creek | Winter | Claw | 3 | -21.61 ± 0.40 | 2.01 ± 1.49 |
| Little-bronze Cuckoo | *Chrysococcyx minutillus* | Partially migratory | Cocoa Creek | Summer | Blood | 1 | -25.30 | 5.31 |
|  |  |  |  |  | Claw | 1 | -22.68 | 4.60 |
| Leaden Flycatcher | *Myiagra rubecula* | Partially migratory | Cocoa Creek | Winter | Blood | 3 | -22.66 ± 0.72 | 4.61 ± 0.45 |
|  |  |  |  |  | Claw | 3 | -20.99 ± 0.50 | 5.51 ± 0.20 |
|  |  |  | Healy Creek | Winter | Blood | 8 | -22.07 ± 0.38 | 4.57 ± 1.86 |
|  |  |  |  |  | Claw | 8 | -20.70 ± 0.49 | 3.50 ± 2.31 |
| Rufous Fantail | *Rhipidura rufifrons* | Partially migratory | Cocoa Creek | Summer | Blood | 1 | -22.41 | 6.18 |
|  |  |  |  |  | Claw | 1 | -21.74 | 5.25 |
|  |  |  |  | Winter | Claw | 1 | -20.41 | 6.70 |
|  |  |  | Healy Creek | Winter | Blood | 1 | -22.32 | 6.33 |
|  |  |  |  |  | Claw | 1 | -20.32 | 6.07 |
| Rufous Whistler | *Pachycephala rufiventris* | Partially migratory | Cocoa Creek | Summer | Blood | 3 | -22.66 ± 1.01 | 6.06 ± 1.30 |
|  |  |  |  |  | Claw | 2 | -18.93 ± 2.17 | 5.69 ± 1.63 |
|  |  |  |  | Winter | Blood | 1 | -22.33 | 6.53 |
|  |  |  |  |  | Claw | 1 | -20.60 | 6.55 |
|  |  |  | Healy Creek | Winter | Blood | 1 | -22.70 | 6.61 |
|  |  |  |  |  | Claw | 1 | -20.85 | 6.18 |
| Shining Flycatcher | *Myiagra alecto* | Sedentary | Cocoa Creek | Summer | Blood | 3 | -21.45 ± 0.54 | 7.40 ± 0.32 |
|  |  |  |  |  | Claw | 2 | -19.75 ± 0.03 | 6.38 ± 0.69 |
|  |  |  |  | Winter | Blood | 3 | -21.48 ± 1.00 | 6.60 ± 1.38 |
|  |  |  |  |  | Claw | 3 | -19.92 ± 0.93 | 6.61 ± 1.25 |
|  |  |  | Healy Creek | Winter | Blood | 3 | -20.68 ± 0.72 | 3.79 ± 1.42 |
|  |  |  |  |  | Claw | 3 | -19.36 ± 0.46 | 3.86 ± 1.46 |
| Spectacled Monarch | *Symposiarchus trivirgatus* | Partially migratory | Cocoa Creek | Summer | Blood | 3 | -23.86 ± 0.99 | 5.55 ± 0.94 |
|  |  |  |  |  | Claw | 3 | -22.06 ± 1.03 | 4.97 ± 0.46 |
|  |  |  |  | Winter | Blood | 4 | -23.68 ± 0.51 | 5.5 ± 0.18 |
|  |  |  |  |  | Claw | 3 | -21.61 ± 0.40 | 5.91 ± 0.17 |
|  |  |  | Healy Creek | Winter | Blood | 1 | -25.07 | 6.56 |
|  |  |  |  |  | Claw | 1 | -23.68 | 6.83 |
| Nectarivore-Insectivores | | | | | | | | |
| Brown-backed Honeyeater | *Ramsayornis modestus* | Partially migratory | Cocoa Creek | Summer | Blood | 2 | -23.83 ± 0.42 | 5.87 ± 0.15 |
|  |  |  |  |  | Claw | 2 | -22.4 ± 0.07 | 6.37 ± 0.44 |
|  |  |  |  | Winter | Blood | 3 | -24.12 ± 0.27 | 5.28 ± 0.34 |
|  |  |  |  |  | Claw | 3 | -22.27 ± 0.38 | 5.93 ± 0.24 |
|  |  |  | Healy Creek | Winter | Blood | 10 | -23.58 ± 0.74 | 4.05 ± 3.91 |
|  |  |  |  |  | Claw | 8 | -22.26 ± 0.83 | 3.58 ± 2.06 |
| Brown Honeyeater | *Lichmera indistincta* | Sedentary and nomadic | Cocoa Creek | Summer | Claw | 1 | -20.34 | 6.74 |
|  |  |  |  |  | Blood | 2 | -23.30 ± 0.29 | 6.74 ± 0.33 |
|  |  |  |  | Winter | Claw | 2 | -21.75 ± 0.23 | 6.75 ± 0.74 |
|  |  |  | Healy Creek | Winter | Blood | 24 | -23.21 ± 0.51 | 4.99 ± 2.09 |
|  |  |  |  |  | Claw | 20 | -21.73 ± 0.51 | 5.34 ± 2.91 |
| Dusky Honeyeater | *Myzomela obscura* | Sedentary and nomadic | Cocoa Creek | Summer | Blood | 17 | -24.35 ± 1.00 | 6.74 ± 0.32 |
|  |  |  |  |  | Claw | 13 | -22.22 ± 0.99 | 6.16 ± 0.32 |
|  |  |  |  | Winter | Blood | 12 | -24.54 ± 0.59 | 6.27 ± 0.66 |
|  |  |  |  |  | Claw | 15 | -22.85 ± 0.43 | 5.85 ± 0.95 |
|  |  |  | Healy Creek | Winter | Blood | 22 | -24.49 ± 0.28 | 5.485 ± 5.64 |
|  |  |  |  |  | Claw | 18 | -22.56 ± 0.35 | 5.47 ± 6.37 |
| Horn-billed Friarbird | *Philemon yorki* | Nomadic | Cocoa Creek | Summer | Blood | 3 | -24.20 ± 0.47 | 7 ± 0.41 |
|  |  |  |  |  | Claw | 3 | -21.29 ± 0.12 | 5.91 ± 0.73 |
|  |  |  |  | Winter | Claw | 1 | -21.49 | 7.15 |
|  |  |  | Healy Creek | Winter | Blood | 4 | -23.75 ± 0.36 | 5.45 ± 1.31 |
|  |  |  |  |  | Claw | 5 | -21.49 ± 0.43 | 5.39 ± 1.23 |
| Olive-backed Sunbird | *Nectarinia jugularis* | Partially migratory | Cocoa Creek | Summer | Blood | 3 | -22.77 ± 0.73 | 7.49 ± 0.17 |
|  |  |  |  |  | Claw | 4 | -21.57 ± 0.66 | 7.84 ± 0.18 |
|  |  |  |  | Winter | Blood | 10 | -22.75 ± 0.67 | 7.31 ± 0.51 |
|  |  |  |  |  | Claw | 10 | -21.51 ± 0.66 | 7.76 ± 0.61 |
|  |  |  | Healy Creek | Winter | Blood | 17 | -23.13 ± 0.99 | 6.72 ± 3.31 |
|  |  |  |  |  | Claw | 16 | -21.42 ± 1.03 | 7.77 ± 3.54 |
| White-throated Honeyeater | *Melithreptus albogularis* | Partially migratory | Cocoa Creek | Summer | Blood | 3 | -23.72 ± 0.13 | 6.26 ± 0.11 |
|  |  |  |  |  | Claw | 4 | -21.91 ± 0.13 | 5.94 ± 0.15 |
|  |  |  | Healy Creek | Winter | Blood | 7 | -23.61 ± 0.67 | 6.09 ± 0.22 |
|  |  |  |  |  | Claw | 7 | -21.89 ± 0.65 | 6.23 ± 0.41 |
| Yellow Honeyeater | *Lichenostomus flavus* | Sedentary | Cocoa Creek | Summer | Blood | 1 | -24.51 | 6.90 |
|  |  |  |  |  | Claw | 1 | -21.93 | 6.07 |
| Yellow-spotted Honeyeater | *Meliphaga notata* | Sedentary and nomadic | Cocoa Creek | Summer | Blood | 9 | -24.47 ± 0.33 | 6.29 ± 0.60 |
|  |  |  |  |  | Claw | 7 | -21.85 ± 0.51 | 5.95 ± 0.38 |
|  |  |  |  | Winter | Blood | 9 | -24.64 ± 0.50 | 6 ± 0.77 |
|  |  |  |  |  | Claw | 9 | -22.62 ± 0.34 | 6.32 ± 0.76 |
|  |  |  | Healy Creek | Winter | Blood | 3 | -23.59 ± 1.36 | 5.57 ± 1.34 |
|  |  |  |  |  | Claw | 2 | -22.10 ± 0.44 | 5.73 ± 1.56 |
| Omnivores | | | | | | | | |
| Great Bowerbird | *Chlamydera nuchalis* | Sedentary | Cocoa Creek | Summer | Blood | 1 | -24.71 | 5.46 |
|  |  |  |  |  | Claw | 1 | -22.29 | 4.50 |
| Mistletoebird | *Dicaeum hirundinaceum* | Nomadic | Healy Creek | Winter | Blood | 5 | -24.94 ± 0.67 | 2.19 ± 1.56 |
|  |  |  |  |  | Claw | 5 | -23.61 ± 0.71 | 2.6 ± 2.51 |
| Olive-backed Oriole | *Oriolus sagittatus* | Partially migratory | Cocoa Creek | Summer | Blood | 1 | -22.05 | 7.07 |
|  |  |  |  |  | Claw | 1 | -20.94 | 6.47 |
| Brush Cuckoo | *Cacomantis variolosus* | Partially migratory | Cocoa Creek | Summer | Blood | 1 | -23.10 | 5.22 |
|  |  |  |  |  | Claw | 1 | -14.43 | 5.19 |
| Cicadabird | *Coracina tenuirostris* | Partially migratory | Cocoa Creek | Summer | Blood | 2 | -24.37 ± 0.03 | 5.37 ± 0.09 |
|  |  |  |  |  | Claw | 1 | -21.70 | 5.39 |
| Little Shrike-thrush | *Colluricincla megarhyncha* | Sedentary | Cocoa Creek | Summer | Blood | 3 | -23.56 ± 0.61 | 5.77 ± 0.65 |
|  |  |  |  |  | Claw | 3 | -21.33 ± 0.77 | 5.57 ± 0.43 |
|  |  |  | Healy Creek | Winter | Blood | 1 | -22.48 | 2.89 |
|  |  |  |  |  | Claw | 1 | -20.48 | 3.62 |
| Spangled Drongo | *Dicrurus bracteatus* | Partially migratory | Cocoa Creek | Summer | Blood | 1 | -23.43 | 6.10 |
|  |  |  |  |  | Claw | 1 | -20.98 | 5.65 |
| Varied Triller | *Lalage leucomela* | Partially migratory | Cocoa Creek | Summer | Blood | 4 | -23.03 ± 0.58 | 6.79 ± 0.68 |
|  |  |  |  |  | Claw | 4 | -21.34 ± 0.64 | 5.88 ± 0.86 |
|  |  |  |  | Winter | Blood | 2 | -22.93 ± 0.37 | 6.7 ± 0.17 |
|  |  |  |  |  | Claw | 2 | -21.09 ± 1.19 | 5.96 ± 1.06 |
|  |  |  | Healy Creek | Winter | Blood | 5 | -23.28 ± 0.76 | 3.28 ± 2.77 |
|  |  |  |  |  | Claw | 6 | -21.19 ± 0.51 | 3.66 ± 2.38 |
| White-bellied Cuckoo-shrike | *Coracina papapuensis* | Partially migratory | Cocoa Creek | Summer | Blood | 1 | -24.19 | 6.17 |
|  |  |  |  |  | Claw | 1 | -21.70 | 5.44 |
| Granivores | | | | | | | | |
| Peaceful Dove | *Geopelia striata* | Sedentary | Cocoa Creek | Summer | Blood | 2 | -18.07 ± 1.81 | 4.89 ± 0.47 |
|  |  |  |  |  | Claw | 1 | -16.38 | 5.04 |
