# Supplementary material for: Stable isotopes reveal opportunistic foraging in a spatiotemporally heterogeneous environment: Bird assemblages in mangrove forests
Source: PLoS One. 2018 Nov 15;13(11):e0206145. doi: 10.1371/journal.pone.0206145 (PMC6237324; doi:10.1371/journal.pone.0206145)
Supplement: S3 Appendix — Table A. δ13C and δ15N values (mean ± sd) and sample size (n) of basal food sources at each sampling site during wet and dry seasons. Sources are grouped by their vegetation type: mangrove, woodland, or saltmarsh. (DOCX) [file pone.0206145.s003.docx]

**S3 Appendix**

**Table A.** **δ^13^C and δ^15^N signatures (mean ± sd) and sample size (n) of basal food sources at each sampling site during wet and dry seasons.** Sources are grouped by their vegetation type: mangrove, woodland, or saltmarsh.

| Site | Source | Season | n | δ^13^C (‰) ± sd | δ^15^N (‰) ± sd | C% concentration ± sd | N% concentration ± sd | C:N ± sd |
| --- | --- | --- | --- | --- | --- | --- | --- | --- |
| Mangrove | | | | | | | | |
| Cocoa Creek | Crabs (*Sesarma* spp.) | Summer | 4 | -21.54 ± 2.04 | 4.80 ± 0.61 | 0.33 ± 0.01 | 0.09 ± 0.01 | 3.5 ± 0.3 |
|  |  | Winter | 5 | 21.33 ± 3.71 | 5.1 ± 1.33 | 0.36 ± 0.07 | 0.10 ± 0.02 | 3.39 ± 0.22 |
|  | Fish (Family Mugilidae, Lutjanidae, Leiognathidae, Sparidae, Gobiidae) | Summer | 6 | -20.51 ± 2.1 | 7.26 ± 0.99 | 0.41 ± 0.03 | 0.12 ± 0.008 | 3.32 ± 0.08 |
|  |  | Winter | 5 | -18.52 ± 0.62 | 6.7 ± 1.78 | 0.39 ± 0.06 | 0.11 ± 0.02 | 3.32 ± 0.07 |
|  | Insects (Order Coleoptera, Lepidoptera, Phasmatodea, Blattodea; and Family Salticidae, Flatidae, Deinopidae, Thomisidae, Formicidae) | Summer | 4 | -25.35 ± 2.36 | 2.94 ± 2.18 | 0.46 ± 0.08 | 0.09 ± 0.03 | 5.56 ± 1.7 |
|  |  | Winter | 5 | 25.04 ± 0.74 | 2.02 ± 2.19 | 0.55 ± 0.08 | 0.11 ± 0.02 | 4.87 ± 0.65 |
|  | Leaves (*Rhizophora* spp.) | Summer | 4 | -28.99 ± 0.23 | 2.57 ± 0.09 | 0.46 ± 0.08 | 0.016 ± 0.001 | 29.01 ± 3.2 |
|  |  | Winter | 5 | -29.45 ± 0.31 | 2.53 ± 0.37 | 0.45 ± 0.09 | 0.008 ± 0.001 | 52.73 ± 7.1 |
| Healy Creek | Crab (*Sesarma* spp.) | Winter | 4 | -19.9 ± 1.32 | -0.58 ± 2.72 | 0.42 ± 0.1 | 0.12 ± 0.03 | 3.29 ± 0.12 |
|  | Fish (Family Gobiidae) | Winter | 4 | -23.48 ± 2.00 | 9.2 ± 0.79 | 0.40 ± 0.08 | 0.12 ± 0.02 | 3.25 ± 0.05 |
|  | Insects (Order Coleoptera, Lepidoptera, Phasmatodea, Blattodea; and Family Salticidae, Flatidae, Deinopidae, Thomisidae, Formicidae) | Winter | 5 | -24.55 ± 1.13 | 1.37 ± 5.68 | 0.43 ± 0.19 | 0.10 ± 0.05 | 4.38 ± 0.4 |
|  | Leaves (*Rhizophora* spp.) | Winter | 5 | -28.90 ± 0.08 | 3.69 ± 1.29 | 0.38 ± 0.06 | 0.01 ± 0.001 | 37.68 ± 5.9 |
| Woodland | | | | | | | | |
| Cocoa Creek | Insects (Order Coleoptera, Lepidoptera, Phasmatodea, Blattodea; and Family Salticidae, Flatidae, Deinopidae, Thomisidae, Formicidae) | Summer | 9 | -24.1 ± 1.9 | 3.86 ± 1.57 | 0.41 ± 0.1 | 0.09 ± 0.02 | 4.43 ± 0.42 |
|  |  | Winter | 10 | 25.08 ± 2.95 | 3.3 ± 2.13 | 0.43 ± 0.1 | 0.09 ± 0.01 | 4.77 ± 0.75 |
|  | Leaves (E*ucalyptus* spp., *Melaleuca* spp.) | Summer | 4 | -29.8 ± 1.66 | -0.08 ± 1.56 | 0.45 ± 0.06 | 0.013 ± 0.004 | 37.78 ± 17.9 |
|  |  | Winter | 10 | -30.8 ± 1.49 | -0.77 ± 0.37 | 0.44 ± 0.09 | 0.009 ± 0.002 | 49.51 ± 6.48 |
| Healy Creek | Insects (Order Coleoptera, Lepidoptera, Phasmatodea, Blattodea; and Family Salticidae, Flatidae, Deinopidae, Thomisidae, Formicidae) | Winter | 5 | -25.50 ± 0.99 | -3.1 ± 2.91 | 0.41 ± 0.14 | 0.08 ± 0.02 | 4.70 ± 0.7 |
|  | Leaves (E*ucalyptus* spp., *Melaleuca* spp.) | Winter | 10 | -30.96 ± 0.35 | -9.84 ± 4.19 | 0.51 ± 0.12 | 0.01 ± 0.005 | 41.75 ± 11.64 |
| Saltmarsh | | | | | | | | |
| Cocoa Creek | Insects (Family Formicidae) and crabs (*Uca* spp*.*) | Summer | 4 | -15.97 ± 0.76 | 7.44 ± 0.56 | 0.41 ± 0.04 | 0.11 ± 0.001 | 3.71 ± 0.4 |
|  |  | Winter | 10 | -15.64 ± 0.85 | 7.45 ± 0.88 | 0.38 ± 0.1 | 0.10 ± 0.02 | 3.64 ± 0.2 |
| Healy Creek | Insects (Family Formicidae) and crabs (*Uca* spp*.*) | Winter | 5 | -11.2 ± 0.3 | -4.5 ± 0.7 | 0.37 ± 0.05 | 0.1 ± 0.7 | 3.48 ± 0.09 |
