# Supplementary material for: Stable isotopes reveal opportunistic foraging in a spatiotemporally heterogeneous environment: Bird assemblages in mangrove forests
Source: PLoS One. 2018 Nov 15;13(11):e0206145. doi: 10.1371/journal.pone.0206145 (PMC6237324; doi:10.1371/journal.pone.0206145)
Supplement: S4 Appendix — Table A. Results of Welch’s two-sample t-tests determining differences in bird blood and claw δ13C and δ15N values between seasons at Cocoa creek. P-values less than, or equal to, 0.05 are highlighted in bold. Table B. Results of Welch’s two-sample t-tests determining differences in source δ13C and δ15N values between seasons at Cocoa creek. P-values less than, or equal to, 0.05 are highlighted in bold. Table C. Results of Welch’s two-sample t-tests determining differences in bird blood and claw δ13C and δ15N values between sites. P-values less than, or equal to, 0.05 are highlighted in bold. Table D. Results of Welch’s two-sample t-tests determining differences in source δ13C and δ15N values between sites. P-values less than, or equal to, 0.05 are highlighted in bold. (DOCX) [file pone.0206145.s004.docx]

**S4 Appendix**

**Table A. Results of Welch’s two-sample t-tests determining differences in bird blood and claw δ^13^C and δ^15^N signatures between seasons at Cocoa Creek.** P-values less than, or equal to, 0.05 are highlighted in bold.

| Comparison | | *t-*test statistic | degrees of freedom | p-value |
| --- | --- | --- | --- | --- |
| Consumer tissues | | | | |
| Tissue |  |  |  |  |
| Blood | Wet season δ13C vs. Dry season δ13C | 0.07 | 129.9 | 0.95 |
|  | Wet season δ15N vs. Dry season δ15N | 0.47 | 108.14 | 0.64 |
| Claw | Wet season δ13C vs. Dry season δ13C | 0.56 | 130.2 | 0.58 |
|  | Wet season δ15N vs. Dry season δ15N | -3.25 | 111.95 | **0.002** |

**Table B. Results of Welch’s two-sample t-tests determining differences in source δ^13^C and δ^15^N signatures between seasons at Cocoa Creek.** P-values less than, or equal to, 0.05 are highlighted in bold.

| Comparison | *t-*test statistic | degrees of freedom | p-value |
| --- | --- | --- | --- |
| Source signatures | | |  |
| Wet season δ13C vs. Dry season δ13C | -0.45 | 96.93 | 0.66 |
| Wet season δ15N vs. Dry season δ15N | 1.99 | 89.3 | **0.05** |

**Table C. Results of Welch’s two-sample t-tests determining differences in bird blood and claw δ^13^C and δ^15^N signatures between sites.** P-values less than, or equal to, 0.05 are highlighted in bold.

| Comparison | | *t-*test statistic | degrees of freedom | p-value |
| --- | --- | --- | --- | --- |
| Consumer tissues | | | | |
| Tissue |  |  |  |  |
| Blood | Cocoa Creek δ13C vs. Healy Creek δ13C | -1.35 | 224.97 | 0.18 |
|  | Cocoa Creek δ15N vs. Healy Creek δ15N | 6.67 | 141.94 | **<0.001** |
| Claw | Cocoa Creek δ13C vs. Healy Creek δ13C | 0.59 | 231.55 | 0.56 |
|  | Cocoa Creek δ15N vs. Healy Creek δ15N | 6.34 | 135.08 | **<0.001** |

**Table D. Results of Welch’s two-sample t-tests determining differences in source δ^13^C and δ^15^N signatures between sites.** P-values less than, or equal to, 0.05 are highlighted in bold.

| Comparison | *t-*test statistic | degrees of freedom | p-value |
| --- | --- | --- | --- |
| Source signatures | | |  |
| Cocoa Creek δ13C vs. Healy Creek δ13C | -0.74 | 74.92 | 0.46 |
| Cocoa Creek δ15N vs. Healy Creek δ15N | 6.49 | 54.57 | **<0.001** |
