# Supplementary material for: Stable isotopes reveal opportunistic foraging in a spatiotemporally heterogeneous environment: Bird assemblages in mangrove forests
Source: PLoS One. 2018 Nov 15;13(11):e0206145. doi: 10.1371/journal.pone.0206145 (PMC6237324; doi:10.1371/journal.pone.0206145)
Supplement: S5 Appendix — Fig A. Simulated mixing regions for source-consumer biplots, categorized by isotope-based foraging group and sampling site. Sources are marked by an ‘x’ and consumers are displayed as black dots. The outer-most contour delineates where 5% of the simulated polygons have a solution (i.e. satisfy point-in-polygon) for each consumer (Smith et al. 2013). Consumers outside of the 95% mixing polygons were removed prior to mixing model analysis (i.e. three consumers in Healy creek isotope group 2 (H_2; d). (DOCX) [file pone.0206145.s005.docx]

**S5 Appendix**

**Fig A.** **Simulated mixing regions for source-consumer biplots, categorized by isotope-based foraging group and sampling site.** Sources are marked by an ‘x’ and consumers are displayed as black dots. The outer-most contour delineates where 5% of the simulated polygons have a solution (i.e. satisfy point-in-polygon) for each consumer (Smith et al. 2013). Consumers outside of the 95% mixing polygons were removed prior to mixing model analysis (i.e. three consumers in Healy creek isotope group 2 (H_2; d).
