# Supplementary material for: Stable isotopes reveal opportunistic foraging in a spatiotemporally heterogeneous environment: Bird assemblages in mangrove forests
Source: PLoS One. 2018 Nov 15;13(11):e0206145. doi: 10.1371/journal.pone.0206145 (PMC6237324; doi:10.1371/journal.pone.0206145)
Supplement: S6 Appendix — Fig A. Plots showing (a) abundance of mangrove bird individuals caught by species, (b) abundance of mangrove bird individuals caught in each literature-based foraging group, and (c) richness of mangrove bird species caught in each literature-based foraging group. Species’ allocation into literature-based foraging groups is shown in Table A2. (DOCX) [file pone.0206145.s006.docx]

**S6 Appendix**


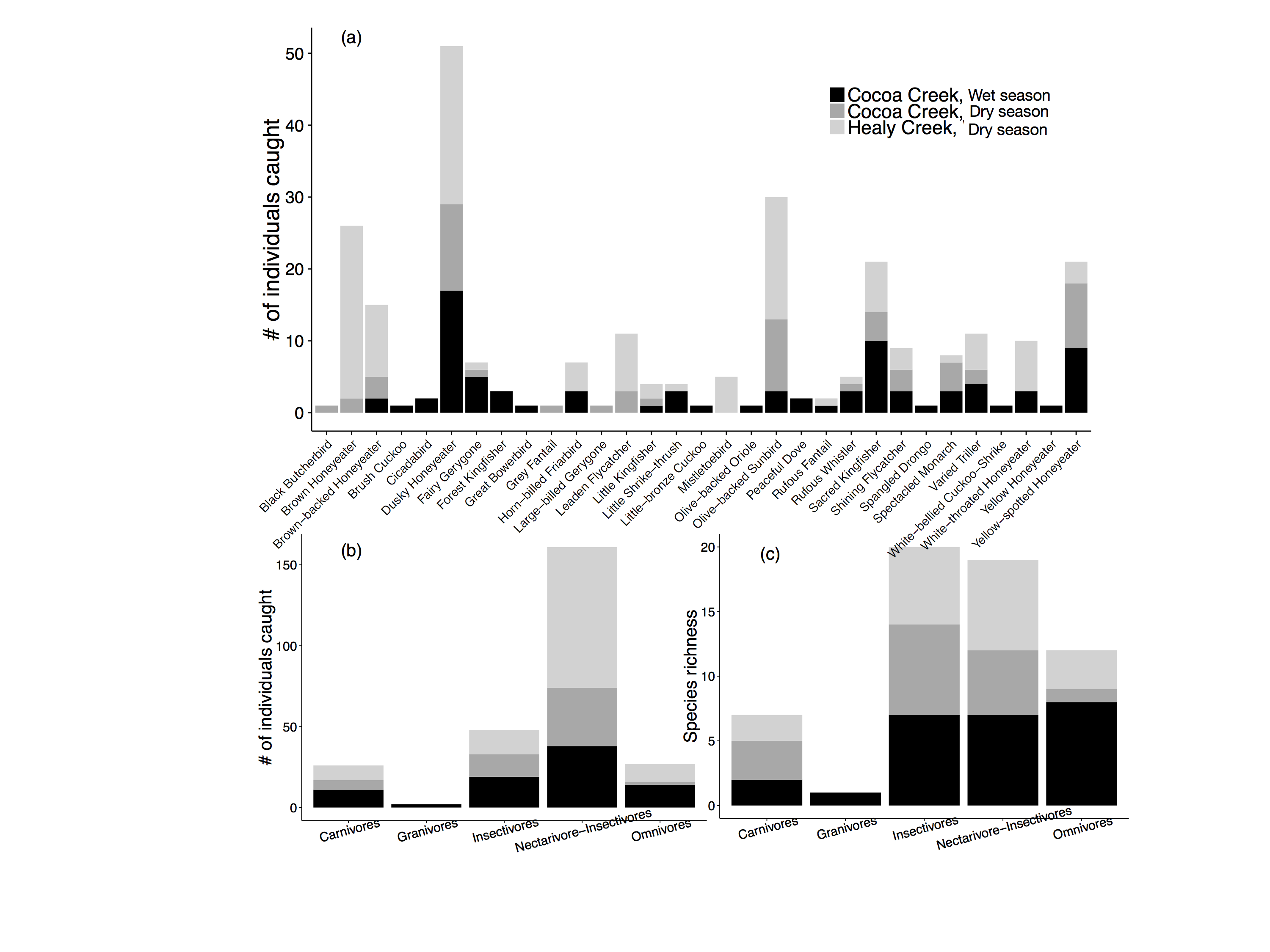


**Fig A. Plots showing (a) abundance of mangrove bird individuals caught by species, (b) abundance of mangrove bird individuals caught in each literature-based foraging group, and (c) richness of mangrove bird species caught in each literature-based foraging group.** Species’ allocation into literature-based foraging groups is shown in Table A2.
