# Supplementary material for: Stable isotopes reveal opportunistic foraging in a spatiotemporally heterogeneous environment: Bird assemblages in mangrove forests
Source: PLoS One. 2018 Nov 15;13(11):e0206145. doi: 10.1371/journal.pone.0206145 (PMC6237324; doi:10.1371/journal.pone.0206145)
Supplement: S7 Appendix — Table A. Pairwise comparisons of standard Bayesian ellipse areas (SEAB) in blood vs. claw tissues of mangrove bird isotope-based foraging groups at Cocoa creek and Healy creek. Probabilities that the SEAB of foraging group claw tissue in rows are smaller than foraging group blood tissue in columns are provided. Highlighted in bold are probabilities that are greater than 0.85 or less than 0.15. Table B. Pairwise comparisons of standard Bayesian ellipse areas (SEAB) of mangrove bird isotope-based foraging groups at Cocoa creek and Healy creek in either blood or claw tissues. Probabilities that the SEAB of foraging groups in rows are smaller than foraging groups in columns are provided. Highlighted in bold are probabilities that are greater than 0.85 or less than 0.15. (DOCX) [file pone.0206145.s007.docx]

**S7 Appendix**

**Table A. Pairwise comparisons of standard Bayesian ellipse areas (SEA_B_) in blood vs. claw tissues of mangrove bird isotope-based foraging groups at Cocoa Creek and Healy Creek.** Probabilities that the SEA_B_ of foraging group claw tissue in rows are smaller than foraging group blood tissue in columns are provided. Highlighted in bold are probabilities that are greater than 0.85 or less than 0.15.

| Site | Tissue | Pairwise comparisons | | | |
| --- | --- | --- | --- | --- | --- |
|  |  |  | Blood | | |
|  |  | p(row<column) | H_1 | H_2 | H_3 |
| Healy Creek | Claws | H_1 | 0.81 |  |  |
|  |  | H_2 |  | **1.00** |  |
|  |  | H_3 |  |  | 0.55 |
|  |  | p(row<column) | C_1 | C_2 | C_3 |
| Cocoa Creek | Claws | C_1 | **0.05** |  |  |
|  |  | C_2 |  | **0.03** |  |
|  |  | C_3 |  |  | **0.02** |

**Table B. Pairwise comparisons of standard Bayesian ellipse areas (SEA_B_) of mangrove bird isotope-based foraging groups at Cocoa Creek and Healy Creek in either blood or claw tissues.** Probabilities that the SEA_B_ of foraging groups in rows are smaller than foraging groups in columns are provided. Highlighted in bold are probabilities that are greater than 0.85 or less than 0.15.

| Site | Tissue | Pairwise comparisons | | | |
| --- | --- | --- | --- | --- | --- |
|  |  | p(row<column) | H_1 | H_2 | H_3 |
| Healy Creek | Blood | H_1 | - | **0.01** | **0.09** |
|  |  | H_2 |  | - | 0.84 |
|  |  | H_3 |  |  | - |
|  | Claws | H_1 | - | **0.07** | 0.54 |
|  |  | H_2 |  | - | **1.00** |
|  |  | H_3 |  |  | - |
|  |  | p(row<column) | C_1 | C_2 | C_3 |
| Cocoa Creek | Blood | C_1 | - | **0.00** | **0.00** |
|  |  | C_2 |  | - | **0.13** |
|  |  | C_3 |  |  | - |
|  | Claws | C_1 | - | **0.00** | **0.00** |
|  |  | C_2 |  | - | 0.53 |
|  |  | C_3 |  |  | - |
