# Supplementary material for: Stable isotopes reveal opportunistic foraging in a spatiotemporally heterogeneous environment: Bird assemblages in mangrove forests
Source: PLoS One. 2018 Nov 15;13(11):e0206145. doi: 10.1371/journal.pone.0206145 (PMC6237324; doi:10.1371/journal.pone.0206145)
Supplement: S8 Appendix — Table A. Individual specialisation metrics calculated from δ13C and δ15N signatures of isotope foraging groups. N = sample size, TNW = Total niche width, WIC = Within-individual component, BIC = Between-individual component, RIS = Relative index of specialisation. (DOCX) [file pone.0206145.s008.docx]

**S8 Appendix**

**Table A. Individual specialisation metrics calculated from δ^13^C and δ^15^N signatures of isotope foraging groups.** N = sample size, TNW = Total niche width, WIC = Within-individual component, BIC = Between-individual component, RIS = Relative index of specialisation.

|  | n | TNW | WIC | BIC | RIS |
| --- | --- | --- | --- | --- | --- |
| Cocoa Creek | | | | | |
| δ^13^C | | | | | |
| C_1 | 37 | 4.18 | 0.36 | 3.82 | 10.62 |
| C_2 | 59 | 0.73 | 0.09 | 0.65 | 7.49 |
| C_3 | 16 | 0.61 | 0.16 | 0.46 | 2.94 |
| δ^15^N | | | | | |
| C_1 | 37 | 1.56 | 0.29 | 1.27 | 4.32 |
| C_2 | 59 | 0.81 | 0.16 | 0.65 | 4.13 |
| C_3 | 16 | 0.93 | 0.37 | 0.56 | 1.50 |
|  |  |  |  |  |  |
| Healy Creek | | | | | |
| δ^13^C | | | | | |
| H_1 | 4 | 4.11 | 0.10 | 4.01 | 39.50 |
| H_2 | 66 | 1.32 | 0.14 | 1.18 | 8.70 |
| H_3 | 22 | 3.76 | 0.08 | 3.68 | 43.60 |
| δ^15^N | | | | | |
| H_1 | 4 | 12.28 | 0.34 | 11.95 | 35.36 |
| H_2 | 66 | 10.59 | 0.57 | 10.03 | 17.73 |
| H_3 | 22 | 8.85 | 0.40 | 8.44 | 20.92 |
