# Supplementary material for: Stable isotopes reveal opportunistic foraging in a spatiotemporally heterogeneous environment: Bird assemblages in mangrove forests
Source: PLoS One. 2018 Nov 15;13(11):e0206145. doi: 10.1371/journal.pone.0206145 (PMC6237324; doi:10.1371/journal.pone.0206145)
Supplement: S9 Appendix — Fig A. Standard Bayesian ellipse areas (SEAB) represent the isotopic niches of mangrove bird species at two mangrove sampling sites. (A) Cocoa Creek and (B) Healy Creek. Line colour and type differentiate between species, and line thickness indicates tissue type (thicker-lined ellipses show blood isotopic niches, and thinner-lined ellipses show claw isotopic niches for each species). Individual consumer isotopic values are also displayed (circles = blood, triangles = claw). Note: ellipse areas of species with samples sizes less than 10 may be underestimated (i.e. Sacred Kingfisher at Healy Creek). Fig B. Standard Bayesian ellipse areas (SEAB) for blood and claw tissues of individual bird species. (A) Cocoa Creek and (B) Healy Creek. Black circles are the mode SEAB, and boxes show the 50%, 75%, and 95% credible intervals. Inset plots show the probability of isotopic niche overlap (mean ± 95% credible intervals) among the bird species. In the inset plots, colour indicates tissue type (‘black’ = blood, ‘grey’ = claw), and symbols represent each species as follows: (triangle) = Olive-backed Sunbird; (square) = Sacred Kingfisher; (circle) = Dusky Honeyeater. Fig C. Relative index of specialisation (RIS) for δ13C and δ15N values of individual bird species. (A) Cocoa Creek and (B) Healy Creek. Black bars = δ13C; Grey bars = δ15N. (DOCX) [file pone.0206145.s009.docx]

**S9 Appendix**

**Fig A. Standard Bayesian ellipse areas (SEA_B_) represent the isotopic niches of mangrove bird species at two mangrove sampling sites.**

(A) Cocoa Creek and (B) Healy Creek. Line colour and type differentiate between species, and line thickness indicates tissue type (thicker-lined ellipses show blood isotopic niches, and thinner-lined ellipses show claw isotopic niches for each species). Individual consumer isotopic values are also displayed (circles = blood, triangles = claw). Note: ellipse areas of species with samples sizes less than 10 may be underestimated (i.e. Sacred Kingfisher at Healy Creek).

**Fig B. Standard Bayesian ellipse areas (SEA_B_) for blood and claw tissues of individual bird species.**

(A) Cocoa Creek and (B) Healy Creek. Black circles are the mode SEA_B_, and boxes show the 50%, 75%, and 95% credible intervals. Inset plots show the probability of isotopic niche overlap (mean ± 95% credible intervals) among the bird species. In the inset plots, colour indicates tissue type (‘black’ = blood, ‘grey’ = claw), and symbols represent each species as follows: (triangle) = Olive-backed Sunbird; (square) = Sacred Kingfisher; (circle) = Dusky Honeyeater.

**Fig C. Relative index of specialisation (RIS) for δ^13^C and δ^15^N values of individual bird species.**

(A) Cocoa Creek and (B) Healy Creek. Black bars = δ^13^C; Grey bars = δ^15^N.
